# Three millennia of lizard and snake consumption by Natufian foragers

**DOI:** 10.1073/pnas.2609354123

**Published:** 2026-08-17

**Authors:** Ma’ayan Lev, Mina Weinstein-Evron, Reuven Yeshurun

**Affiliations:** ^a^ School of Archaeology and Maritime Cultures, Zinman Institute of Archaeology, University of Haifa, Haifa 3103301, Israel; ^b^Archaeology Stable Isotope Laboratory, Institute for Prehistoric and Protohistoric Archaeology, University of Kiel, Kiel 24118, Germany

**Keywords:** Paleolithic diet, Natufian, squamate, Levant, terminal Pleistocene

## Abstract

Squamate (lizard and snake) exploitation provides a rarely documented window into human dietary flexibility at the end of the Pleistocene. This study analyzes over three millennia of squamate remains from el-Wad Terrace (Israel), revealing that large-bodied snakes and lizards formed a substantial and consistent part of Natufian subsistence. Repeated evidence of butchery on snake vertebrae indicates regular consumption across all occupation phases. Shifts in squamate abundance and bone modification patterns correspond to shifts in site-use intensity and the relative contribution of human and animal predators to archaeofaunal deposition. Species-based habitat weighting indicates a broadly stable Mediterranean maquis environment, where detected environmental shifts would not substantially alter local squamate abundance, demonstrating that variation in squamate use is unlikely to have resulted from environmental forcing. In the regional record, Natufian herpetofaunal assemblages cluster together, demonstrating human foraging choices and contrasting with those of older Paleolithic sites. In the latter, herpetofauna is far less abundant, taxonomically structured by geography, and includes more diverse and smaller-bodied taxa. Striving to understand the socioeconomic circumstances that contributed to relying on squamates for food, we assess their prey ranking by accounting for body size, agility, and the risks associated with handling different taxa. We found that high- and medium-ranked species were consistently targeted despite fluctuations in habitation intensity, suggesting squamate resilience. These results uncover the long-term role of reptiles in emerging sedentary lifeways and possibly early human impact on local reptile communities.

## Significance

Our study reveals a previously underexplored dimension of human–squamate relationship in prehistory. We present direct long-term archaeological evidence for regular butchery and consumption of squamates, documented consistently over millennia at the Natufian site of el-Wad Terrace. Additionally, we provide integration of both lizards and snakes into prey-rank models, offering a more complete framework for understanding the socioeconomic circumstances of incorporating squamates in human diets. Our results demonstrate that reptile exploitation was an established and persistent dietary practice and that squamate consumption intensity is linked to site occupation intensity and emerging sedentism. Regional comparison further shows that Natufian squamate assemblages differ from earlier periods, suggesting that early sedentary communities developed distinct reptile exploitation practices.

**Fig. 1.**
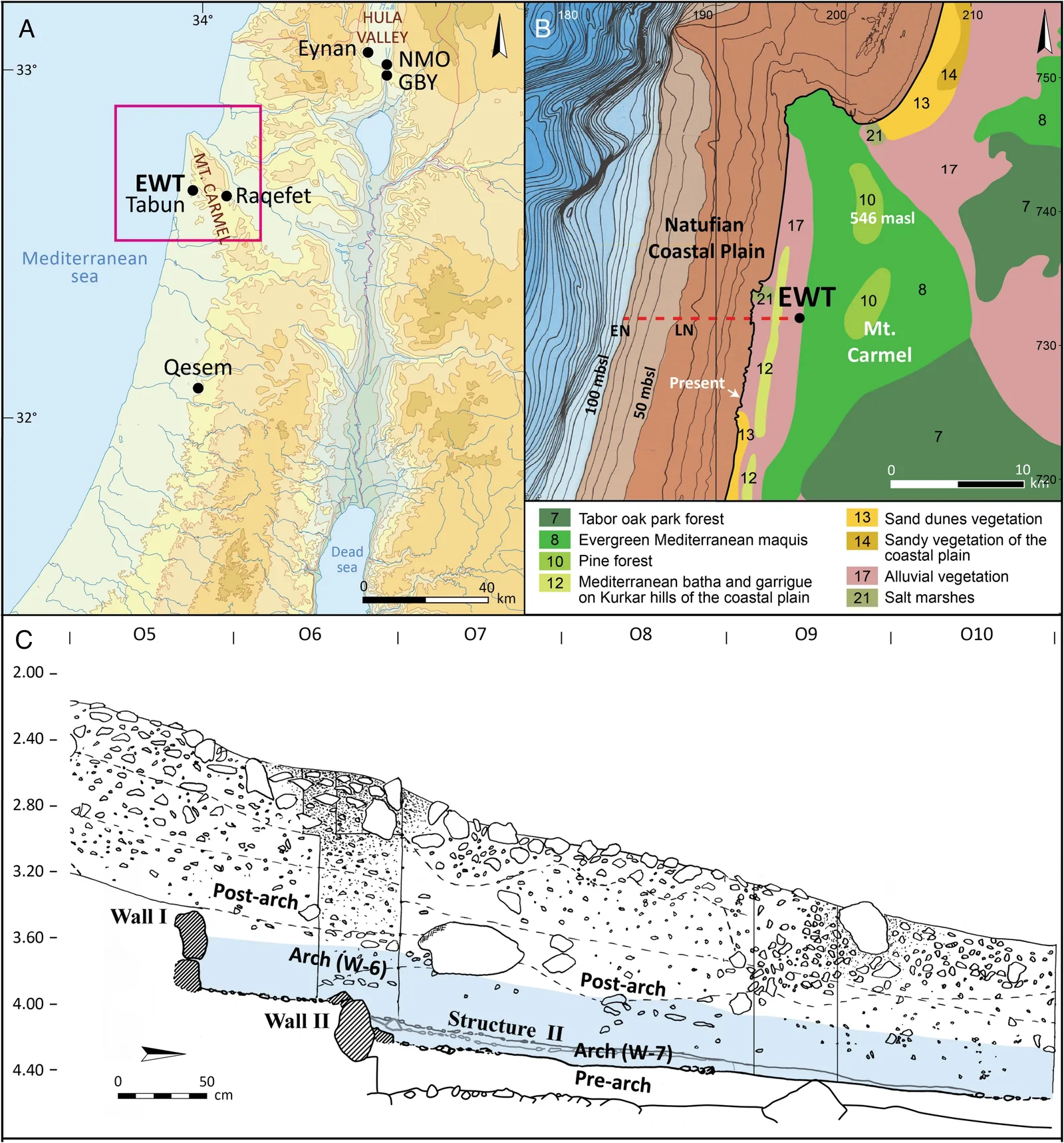
The case study and its regional context. (*A*) Map of the southern Levant showing the locations of EWT and sites mentioned in the comparative analysis. (*B*) Close-up on the Mount Carmel region, showing the present-day vegetation and topography, and the reconstructed Early and Late Natufian (EN and LN) shorelines. (*C*) A simplified stratigraphic profile of the EWT Natufian deposits. The architectural stage (“Arch”) is shaded.

While ethnographic literature demonstrates that lizards and snakes (Order Squamata) are a regular component in the diets of many small-scale human societies, primarily in tropical or warm desert environments (e.g., ref. 1–3), archaeology has paid little attention to the topic (*SI Appendix*, *Squamates in Past and Present Human Diets*). Consequently, it is unclear under what environmental and socioeconomic conditions past human societies consumed squamates, and what part of the diet they constituted. In this study, we explore this topic by analyzing a large sample of herpetofaunal remains from el-Wad Terrace (EWT), Mount Carmel, Israel, one of the most detailed Natufian (ca. 15,000 to 11,700 cal BP) habitation sequences (Fig. 1).

Natufian sites mark a significant episode in human evolution. They exhibit intensifying site-occupation and foraging activity, alongside growing social complexity and initial sedentism. This expanding cultural complexity is manifested in the proliferation of durable architecture, cemeteries, bedrock features, ground stone implements, art items, and broad-spectrum foraging (4–9). Generally speaking, the Natufian subsistence economy relied on intensive hunting and gathering of a broad spectrum of resources, focusing on small-bodied ungulates and small game. It featured a decrease in high-ranking prey and a regular focus on harder-to-get low-ranking prey, presumably due to the increasingly permanent nature of site occupation (10, 11). Squamates present considerable variation in body size, agility, and capture risk, but remain largely excluded from such prey-ranking frameworks, limiting our ability to fully reconstruct prey choice in the early sedentary communities of the latest Pleistocene, and calling for testing squamate–human associations through time in high-resolution sequences.

Significantly, lizard and snake skeletal remains are prevalent in Natufian sites, accounting for up to 40% of the faunal assemblages’ number of identified specimens (NISP; e.g., ref. 12). Previous research established that large-bodied lizards and colubrid snakes were likely consumed in the Early Natufian (EN) occupation of EWT (13) and the Final Natufian habitation at Eynan (14), calling for a more comprehensive study of Natufian squamate exploitation through this culture’s entire duration and various habitation modes. Our present findings originate in 20 stratified layers of Natufian occupation at EWT, representing four distinct stages of site habitation, spanning ca. 15,000 to 11,700 cal. BP. An initial settlement in the prearchitectural stage was followed by a series of built dwellings in the architectural stage, when habitation intensity and sedentism peaked. A postarchitectural stage demonstrating a return to a less permanent mode of site occupation at the end of the EN was then apparent, followed by similar Late Natufian (LN) deposits. This sequence likely comprises a complete demographic cycle of growth, Malthusian equilibrium, collapse, and reorganization, nestled in a resource-rich Mediterranean vegetation zone with hot, dry summers and cool, rainy winters [(15); *SI Appendix*, *The Archaeological Case-Study*].

## Results

**Fig. 2.**
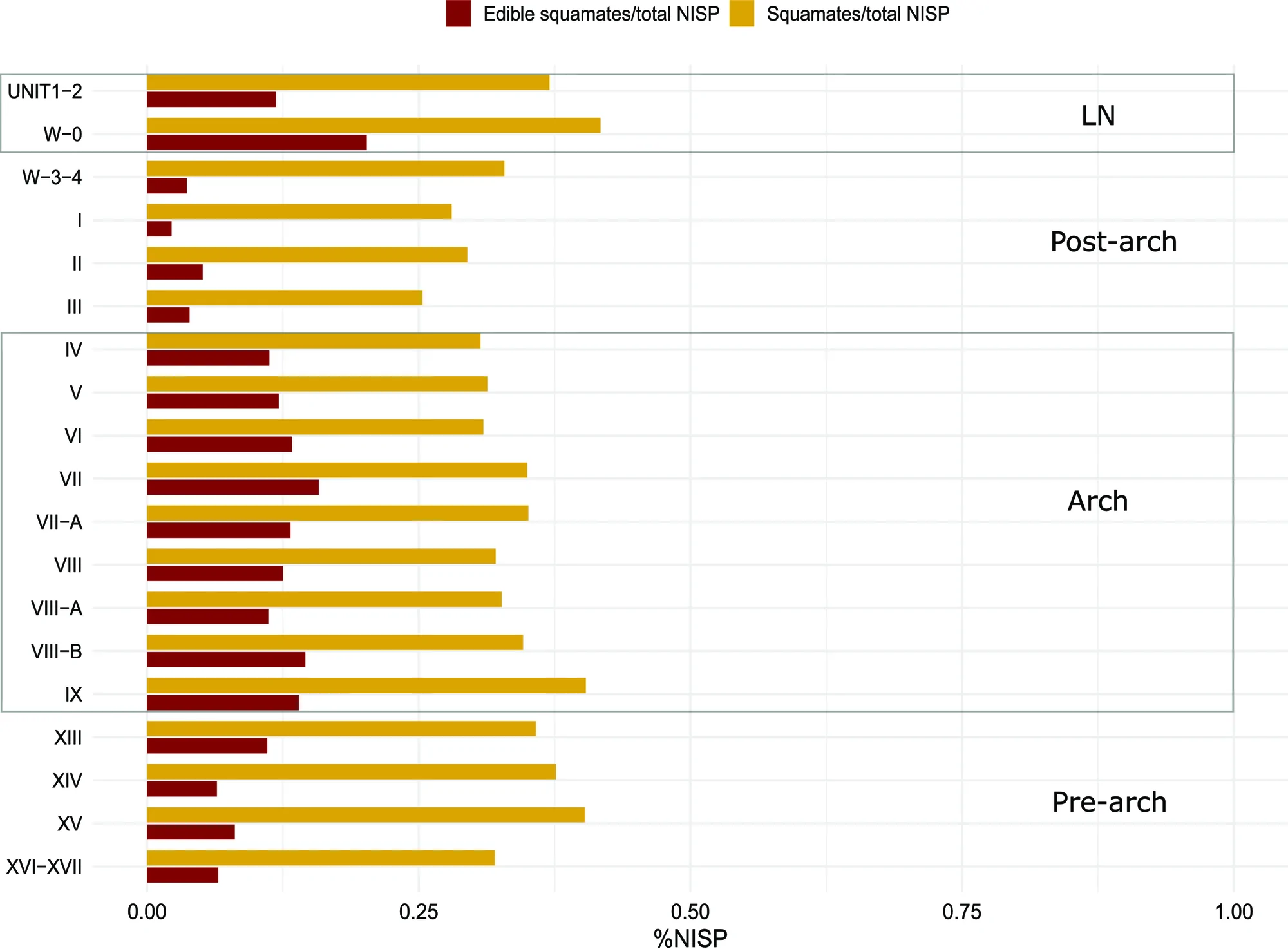
Proportional abundance of squamates and edible squamates in the EWT sequence. based on the assemblages of the 5 mm mesh, which are representative of the macrofauna NISP. Data are from *SI Appendix*, Table S1.

Herpetofauna remains are strikingly abundant in all 20 stratified Natufian samples at EWT. However, this applies primarily to squamates; comparatively, anurans (frogs and toads) are remarkably rare (%NISP = 1) and will thus not be discussed further. According to assemblages produced by the 5 mm mesh, which are representatives of the site’s macrofauna, squamate remains comprise 25 to 42% of the NISP, frequencies similar to those of small ungulates and tortoises (*SI Appendix*, Table S1). Typically, more than one-third of the remains associated with Natufian food consumption belong to lizards and snakes. Still, given the likelihood that some of the squamate remains were introduced by nonhuman agencies [(13), and see below], we distinguish a category of edible squamate remains. It consists of nondigested large (Greatest Length >5 mm) squamate vertebrae, representing specimens that would have been preferentially hunted by humans and were not digested by animals. The size of this category fluctuates greatly, comprising 12 to 17% of the prearchitectural stage NISP, rising to 16 to 24% in the architectural stage, dropping to 3 to 8% in the postarchitectural stage, and rising again to 19 to 35% in the LN stage (Fig. 2). We now turn to explore the taxonomic and taphonomic variability of these taxa along the EWT sequence, using the assemblages produced by both 5 mm and 1 mm meshes.

### Taxonomic Composition.

**Fig. 3.**
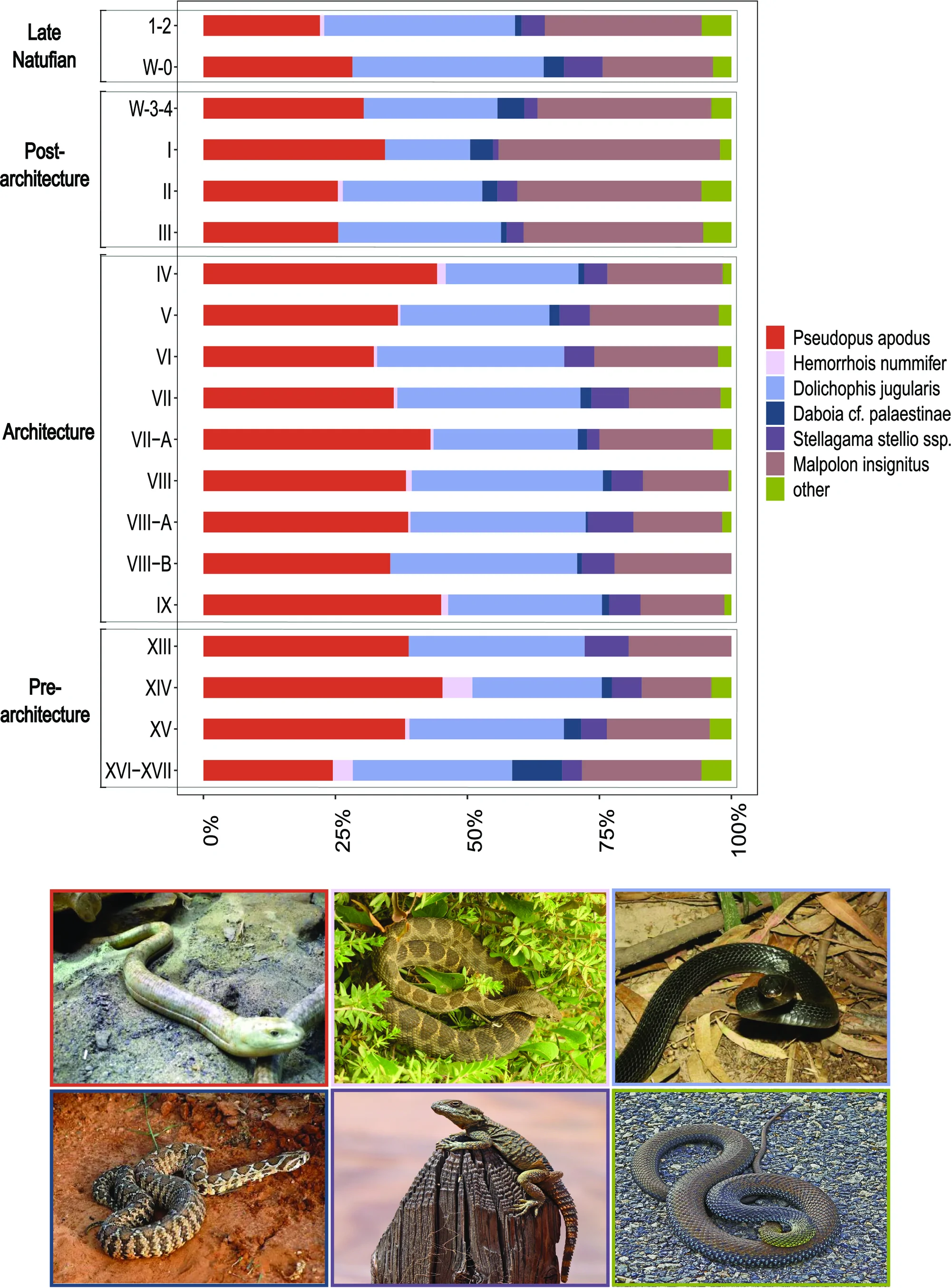
Relative abundances (%NISP) and distribution of the six most common squamate species in EWT. The species photographs (all from WikiCommons, released with CC BY-SA license, credit in parentheses) are, from top left corner in a clockwise order: the European glass lizard, *P. apodus* (Tim Vickers); coin-marked snake, *H. nummifer* (Avi Nahmias); large whip snake, *D. jugularis* (Václav Gvoždík); the eastern Montpellier snake, *M. insignitus* (Juan Lacruz); roughtail rock agama, *S. stellio* (Charles J. Sharp); and the common viper, *D. palaestinae* (Arbel Levy).

The European glass lizard is the most common squamate in the assemblage (*Pseudopus apodus*, NISP = 975, 35% of the total identified species), followed by the large whip snake (*Dolichophis jugularis*, NISP = 881, 32%) and the eastern Montpellier snake (*Malpolon insignitus*, NISP = 626, 22%). Trailing further behind are the roughtail rock agama (*Stellagama stellio* ssp., NISP = 155, 6%), the common viper (*Daboia* cf. *palaestinae*, NISP = 54, 2%), and the coin-marked snake (*Hemorrhois nummifer*, NISP = 22, 1%). While this taxonomic composition is largely consistent across all samples, their relative abundances fluctuate (Fig. 3 and *SI Appendix*, Table S2). Thus, the frequency of the European glass lizard is significant in the prearchitectural (25 to 45% of squamates) and architectural stages (32 to 45%) and slightly lower in the postarchitectural (25 to 34%) and LN stages (22 to 28%). The eastern Montpellier snake presents a somewhat complementary trend: It is moderately abundant in the prearchitectural (13 to 23%) and architectural stages (16 to 24%), becomes more frequent in the postarchitectural stage (34 to 42%), and decreases again in the LN stage (21 to 33%). The large whip snake’s abundances are more stable, comprising 25 to 33% of squamates in the prearchitectural stage, 25 to 36% in the architectural stage, 16 to 31% in the postarchitectural stage, and 36% in the LN stage.

**Fig. 4.**
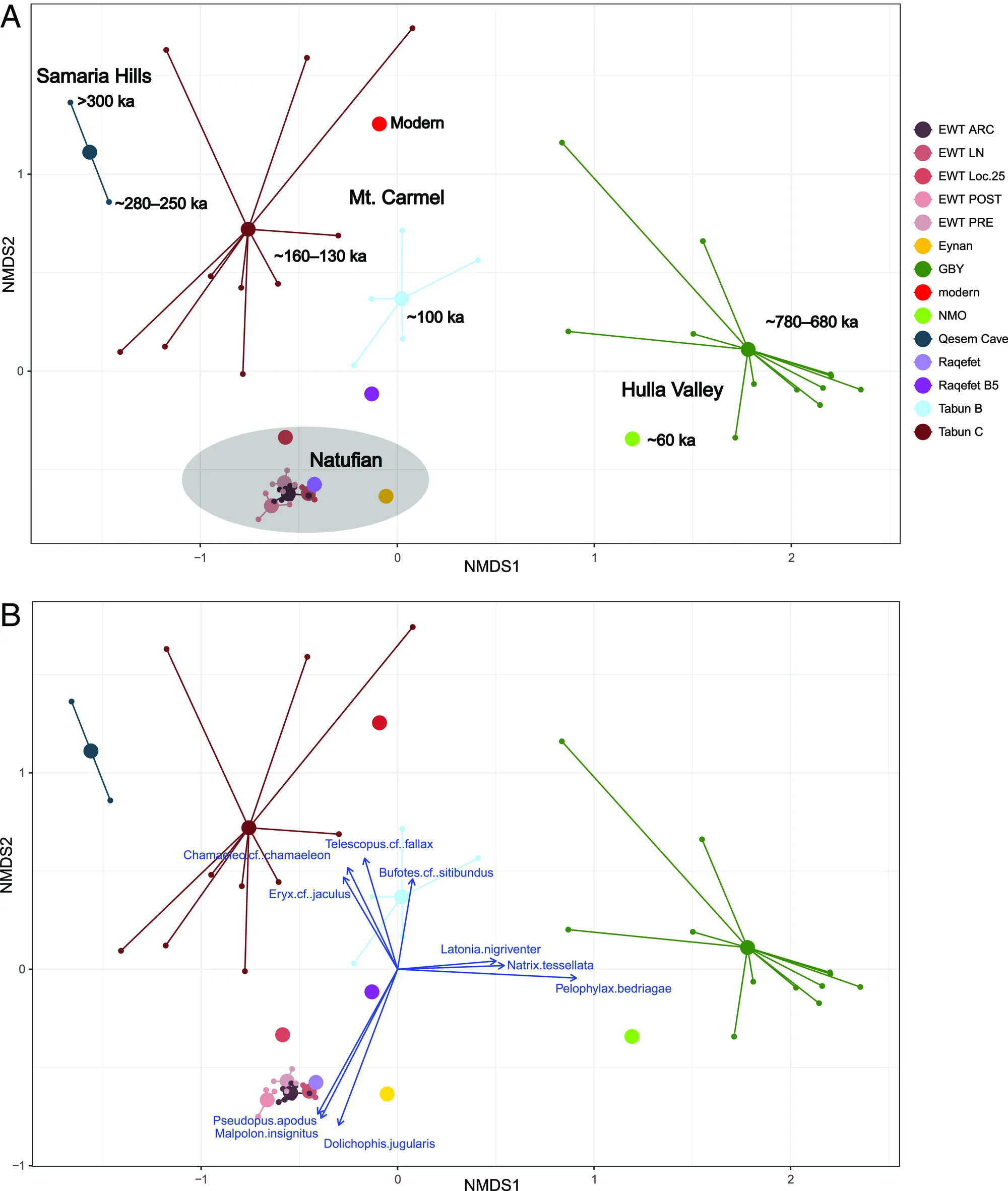
Nonmetric multidimensional scaling (NMDS). (*A*) NMDS of the sampled levels (small circles) and averages (big circles) expressed as Bray–Curtis dissimilarities. Stress level of 0.11. (*B*) NMDS with species vectors (blue arrows) showing the 10 taxa most strongly correlated with the ordination axes.

The taxonomic composition of the EWT samples is assessed by comparing to the full herpetofaunal regional record that includes Natufian samples from two other sites, older Paleolithic remains from four sites, and a recent owl pellet assemblage. We examine whether the Natufian records diverge from other paleoherpetofaunas due to the presumed consumption. Permutational multivariate ANOVA indicates significant differences between the assemblages (PERMANOVA *F* = 9.04, *P* < 0.05). The NMDS plot (Fig. 4*A*) shows that all non-Natufian samples cluster in two groups according to geographic location, either west or east of the main water divide of Israel: Mount Carmel and Samaria Hills to the left and the Hula Valley to the right. Notably, despite being deposited hundreds of thousands of years apart, chronological position seems to have had no effect. Conversely, the Natufian samples plot separately and very close together despite spanning distinct regions (EWT and Raqefet from Mount Carmel and Eynan from the Hula Valley). Still, among the Natufian samples, those of EWT cluster more tightly together, followed by other Natufian sites and mostly far apart from the earlier Tabun Cave assemblages and the recent EWT owl pellet assemblage, all originating within ca. 100 m in the Carmel. The nondomestic, natural accumulations of EWT Locus 25 and Raqefet B5 do not cluster tightly with the Natufian samples either. Overlaying the NMDS with the species vectors (Fig. 4*B*) demonstrates that the Natufian assemblages are primarily distinguished by their association with the large-bodied taxa: *P. apodus*, *M. insignitus,* and *D. jugularis*.

### Taphonomy.

**Fig. 5.**
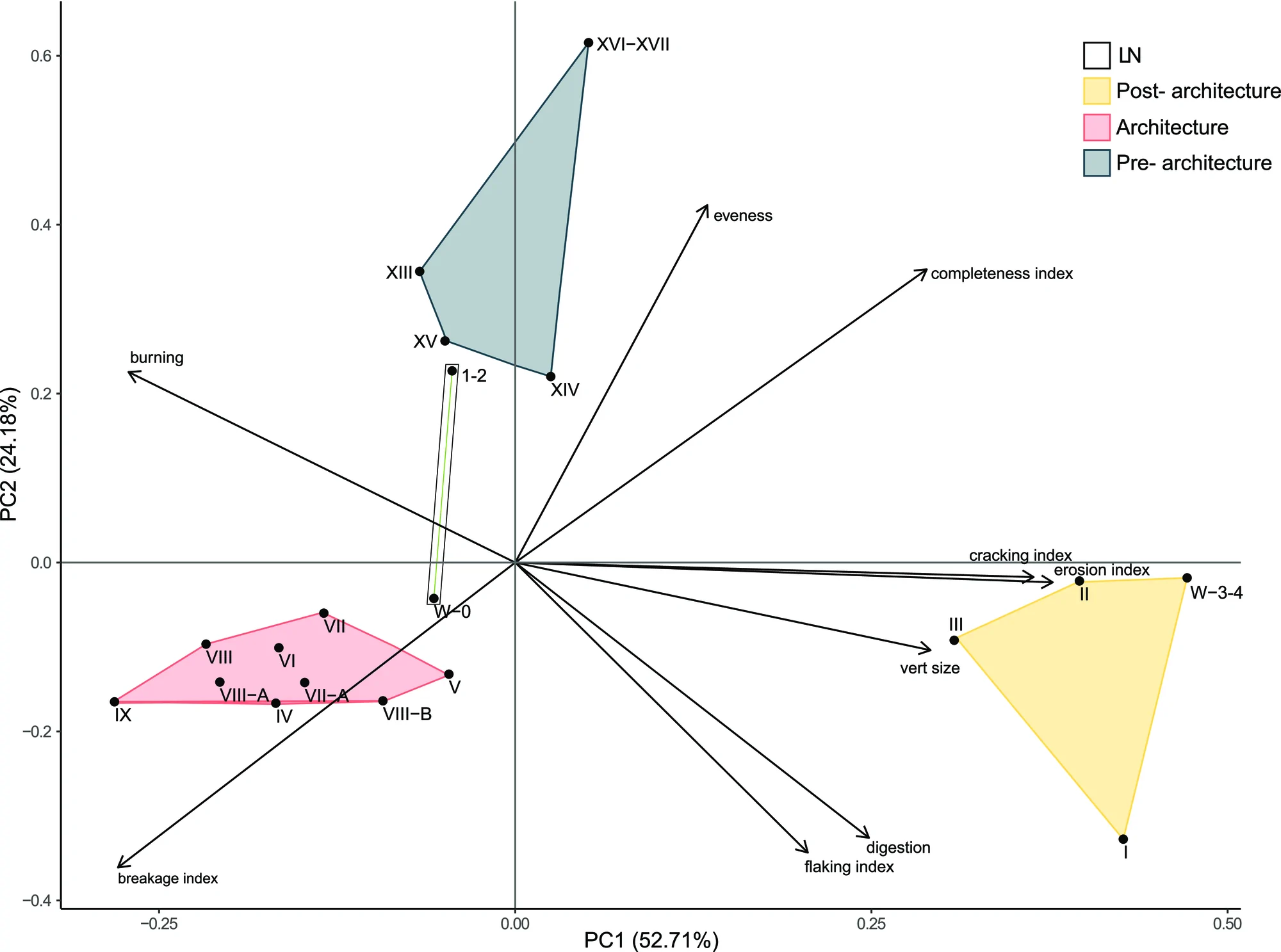
Results of PCA (PC 1 and 2) of the taphonomic indices.

Digestion, burning, cracking, and flaking bone modifications were noted in all EWT squamate samples, but in varying degrees; bone sizes and degrees of fragmentation considerably fluctuated, too (*SI Appendix*, *Extended Taphonomic Analysis*). We performed a principal component (PC) analysis (PCA) of all taphonomic indices except for % cutmarks; the latter were too few per level to allow for intersample comparison (*SI Appendix*, Table S3). The PCA distinguishes clusters within EWT samples, which match almost perfectly the site’s four-stage stratigraphic sequence (Fig. 5). The first PC accounts for 53% of the variance and differentiates the postarchitectural stage; it is primarily associated with high digestion, cracking, and erosion indices, underscoring the considerable contribution of predation and protracted burial to the formation of this stage’s assemblage. The higher frequency of complete bones and the relative paucity of burnt bones further suggest that this stage was characterized by a relatively low habitation intensity. The second PC accounts for 24% of the variance and distinguishes the prearchitectural stage. This distinction is mainly attributable to the high frequency of burnt bones, considerable taxonomic evenness, a relatively large proportion of complete bones, and few digestion modifications, suggesting that nonpredatory (natural) deaths had a greater input in this stage than in others. The levels of the architectural stage feature EWT’s most fragmented squamate assemblage, as reflected by the strong association of the breakage index with this cluster. It also features low taxonomic diversity, relatively few digestion modifications, and a high frequency of burnt bones. The taphonomic signature of the architectural stage attest to repeated trampling and fire use, selection for larger reptiles, and decreased predator activity, thus reflecting the highest habitation intensity in the site’s sequence (as also shown by multiple proxies, ref. 15), with the breakage index tracking shifts in occupation intensity throughout (*SI Appendix*, Fig. S2*C* and Table S3).

**Fig. 6.**
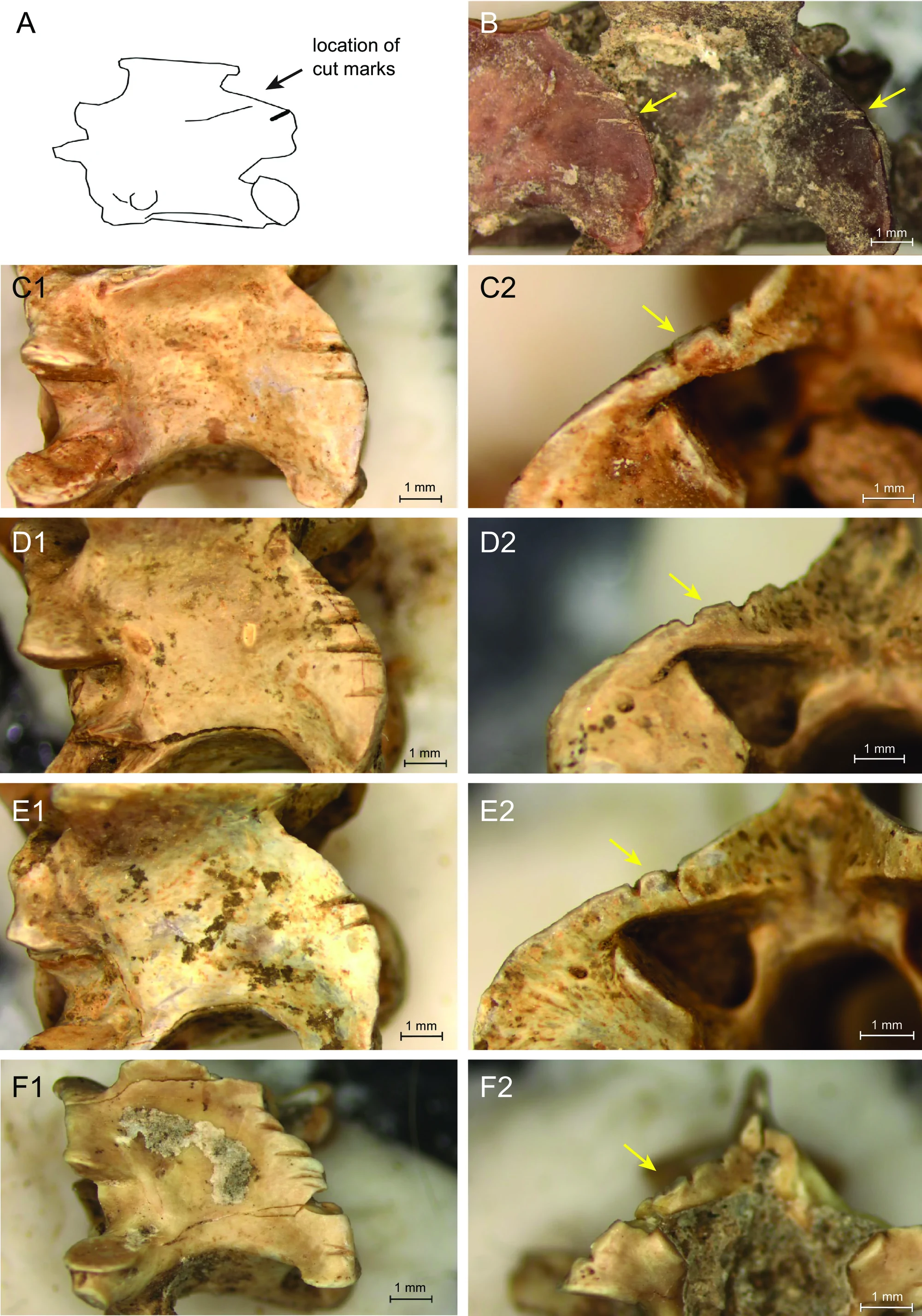
Examples of cutmarks on snake bones at EWT. (*A*) schematic drawing of the cutmark location on the snake vertebrae. (*B*) cutmarks on articulated vertebrae of *M. insignitus*, EWT#24524 to 24525. (*C*) *D. jugularis,* EWT#3261. (*D*) *D. jugularis*, EWT#24513. (*E*) *D. jugularis,* EWT#24516. (*F*) *Colubridae indet*., EWT#24517. In *C*–*F*, 1 is the left lateral-dorsal view, and 2 is the posterior view.

The most telling indication of human manipulation of squamates is provided by cutmarks on snake bones, short linear marks with a V-shaped cross-section. These marks were observed on 82 specimens, all of which occur on the lateral left postzygapophyses of large snakes’ trunk vertebrae (Greatest Length >5 mm; Fig. 6 and *SI Appendix*, Table S4). Often, multiple striations corresponding to a repetitive cutting action are observed on the same specimen. The anatomical and morphological regularity of these marks and their consistent distribution along EWT’s Natufian sequence provide direct evidence that large snakes were butchered.

Regarding nonpredatory (natural) mortality at the site, no complete or semicomplete squamate skeleton was recovered in any of the phases. While some instances of anatomical articulation were observed and could suggest in situ death, the presence of articulated snake vertebrae with cut marks (specimens #24524 to 24525, Fig. 6*B* and specimens #8843 to 8844) indicates that at least some articulated remains had undergone human processing.

### Habitat Weighting Analysis.

**Fig. 7.**
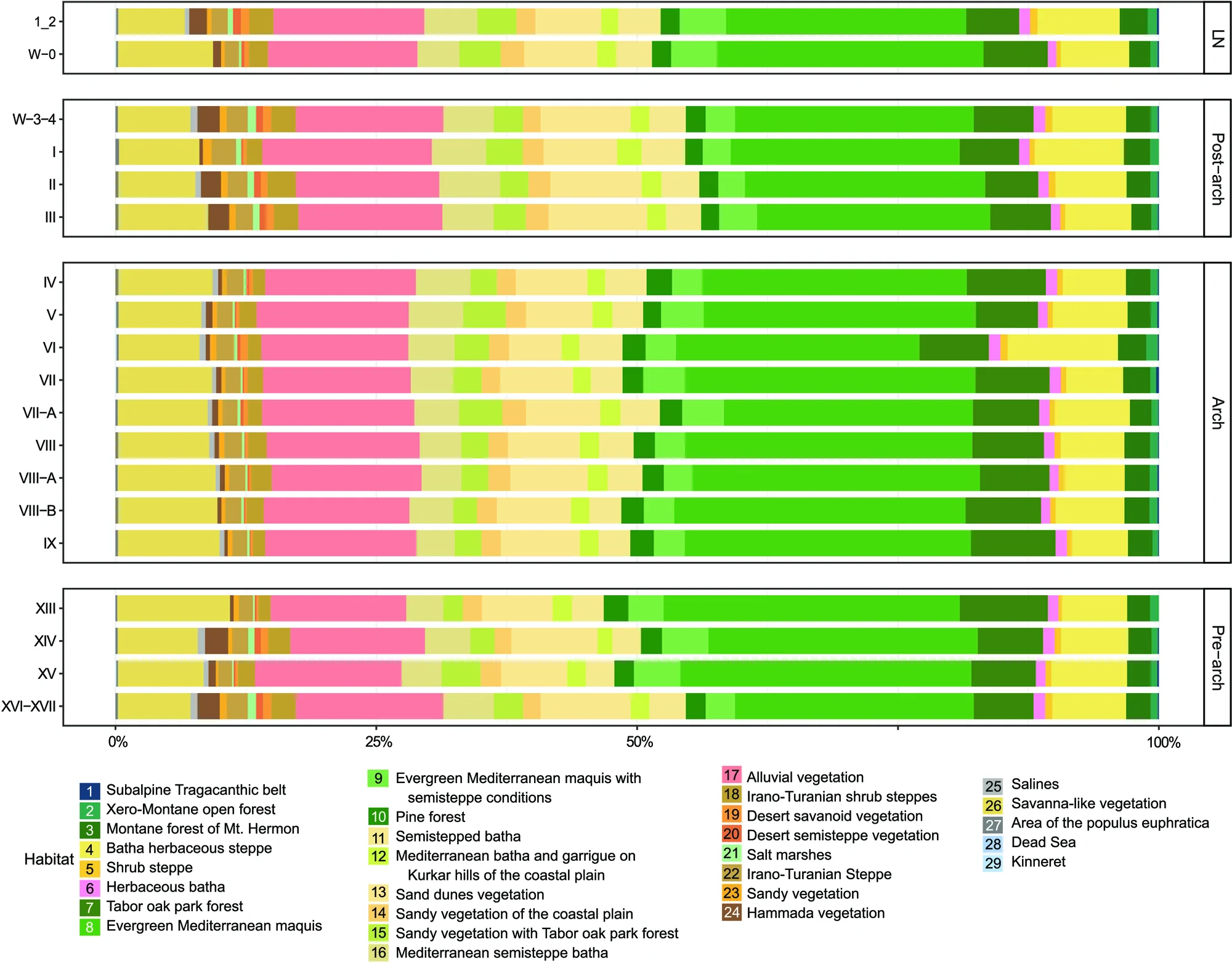
Habitat weighting for the different levels of EWT, divided by stages.

Habitat weighting analysis was performed for each of the EWT levels, which were found to exhibit the same habitat composition throughout with minor variations in their proportional representation (Fig. 7 and *SI Appendix,* Table S5). The Mediterranean maquis habitat is dominant in all samples (22 to 28%), notwithstanding a slight decrease in the postarchitectural stage (22 to 23%). Nonarboreal, open Mediterranean habitats (batha and garigue, including Habitats 4 to 6, 11 to 12, and 16) are also common in all phases (15 to 23%) with peaks in the architectural (Level VI; 23%) and postarchitectural stages (Levels I–II; 20% and 22%). Other Mediterranean habitats, including Mediterranean alluvial vegetation (Habitat 17; 13 to 16%) and the Tabor-oak park forest (Habitat 7; 6 to 8%), are also well represented, as are coastal sandy habitats (Habitats 13 to 15, 10 to 15%). The various open and dry habitats are relatively common in all the phases, predominantly the savannah-like vegetation (Habitat 26; 7 to 11%).

### Prey-Rank Evaluation of Squamate Taxa.

The full prey-rank evaluation of the six main species identified at EWT is presented in *SI Appendix,* Table S6. Among these, the glass lizard ranks highest, being large-bodied, relatively slow-moving, diurnal, and nonvenomous. The large whip snake ranks medium-high, its large body size and diurnal activity are partially offset by its speed and strong bite. The eastern Montpellier snake ranks medium, its mildly venomous and aggressive behavior offsetting its large size. The starred agama, common viper, and coin-marked snake all rank low, due to smaller body size, high risk, and nocturnal activity and aggressiveness, respectively.

## Discussion

The paucity of archaeological evidence for squamate consumption is striking, given the numerous accounts for such behavior among small-scale societies in warm regions (roughly between 30°N and 30°S), which tend to occur in settings with relatively limited seasonality. By contrast, our case study derives from the Mediterranean climate region (32°40′N), where seasonal variation is far more pronounced, highlighting the distinctive context in which Natufian practices unfolded. As noted above, only a handful of case studies worldwide have addressed the topic, almost certainly due to a lack of attention rather than insufficient evidence. Our analysis of the Natufian EWT sequence now demonstrates the butchery, consumption, and deposition of squamates in great detail, allowing us to offer some important insights regarding patterns of selection of snakes and lizards in this early sedentary society.

Our analyses found that several agents were responsible for the accumulation and formation of squamate remains in EWT. These primarily consisted of animal predation and human consumption, which operated against the backdrop of natural deaths and various postdiscard modifications. Animal predation appears to have been more prevalent during the less intensely occupied, postarchitectural stage, whereas human squamate consumption was more significant during the other stages. The squamate remains were deposited in the living areas (e.g., refs. 16 and 17) and underwent characteristic postdiscard damage, whether charring and trampling in the architectural stage or prolonged exposure on the surface in the postarchitectural stage.

The taphonomic and taxonomic analyses of the squamate vertebrae provide direct and indirect evidence of human consumption. The former comprises cutmarks in a consistent anatomical location, spanning the site’s entire occupational sequence, demonstrating that the site’s inhabitants systematically processed squamates. The absence of such cutmarks on glass lizard vertebrae may be due to its much harder skin, which is composed of osteoderms and requires a different butchery technique. Our claim that squamates were purposefully and systematically consumed is also supported by multiple lines of indirect evidence, both at the studied site and the regional record. First, at EWT low-diversity large-squamate assemblages have been recovered in association with consumption refuse consisting of macromammal, water birds and game birds, and tortoise remains rather than natural accumulations of small herpetofauna and micromammal remains (13). Their postdiscard burning and trampling patterns also align with those of unequivocally anthropogenic Natufian refuse (15). Second, the tight clustering of Natufian assemblages across geographically distinct regions, in contrast to earlier assemblages that group by geographic location, supports a shared pattern of species selection that transcends local habitat variation. This is further supported by the dominance of large-bodied species (glass lizard, large whip snake, and the eastern Montpellier snake) in the Natufian assemblages, distinguishing them from earlier Paleolithic sites and modern-day pellet remains in the region. Third, squamates comprise an immense portion of Natufian faunal assemblages: 17% of NISP at Hof Shahaf (18), 25 to 42% at EWT (this study), and 54% at Raqefet Cave in Mount Carmel (19). Conversely, they constitute only 1% of NISP in Middle Paleolithic Misliya Cave, located ca. 7 km north of el-Wad in a similar environment (20), 3% in the natural pitfall trap assemblage of Middle Paleolithic Tabun B, adjacent to EWT (21), and 7 to 14% in the Upper Paleolithic sequence of Manot Cave in the Galilee (22).

While snakes and lizards were consumed throughout the EWT Natufian habitation sequence, some temporal variations are apparent. First, the proportion of edible squamates, those reptiles that would have been preferentially hunted by humans and do not bear signs of animal digestion, is highest in the architectural stage, when the site was most intensely inhabited. Their abundance decreased during the postarchitectural stage, when the site was inhabited seasonally and for shorter durations (15); the postdepositional modifications observed on the squamate remains indicate lengthier exposure to the elements and slower burial, contrasting with more intense trampling, breakage, and burning in the architectural stage. Thus, our results suggest that squamate exploitation positively correlated with habitation intensity, at least during the EN. The meager squamate proportions in pre-Natufian case studies from the region (see above) further support the association of squamate exploitation with increasing sedentism in the Levant. In addition to the general decrease in squamate exploitation following the EN architectural stage, we found a higher relative abundance of the glass lizard in the prearchitectural and architectural stages and an increasing abundance of the eastern Montpellier snake in the postarchitectural stage, while the large whip snake’s abundances remain relatively stable throughout.

Shifts in site habitation intensity may work in tandem with environmental modifications that alter the abundances of certain squamates. Our habitat weighting analysis found no drastic environmental shifts during the site’s sequence, though some variation is detectable. The postarchitectural stage features a slight and temporary decrease in Mediterranean maquis and an increase in open habitats (batha and sandy habitats). Our anthracological analysis suggested the same pattern, featuring a dip in evergreen oak in the postarchitectural stage and a recovery in the LN (23). While we cannot rule out that these environmental fluctuations contributed to changes in the site’s overall habitation intensity, they are unlikely to cause meaningful shifts in the abundance of the main squamate species around the site, given their broad and overlapping habitat preferences.

Consequently, the variations in herpetofauna taxonomy and taphonomy documented here most likely reflect changes in human foraging practices. Given that three species dominate all levels of the site and are all relatively large, their relative ranking is perhaps best assessed with reference to their flight behavior and risk to humans, as detailed in *SI Appendix*, Table S6. Generally, lizards rank higher than snakes because they have comparatively undeveloped predator-avoidance strategies, such as hiding and body armor, and are therefore easier to catch (24). By the same token, snakes rank lower because they are more agile and have relatively developed predator avoidance strategies, including their capacity to inflict venomous or nonvenomous bites, and therefore require more effort to catch. In particular, the glass lizard is a nonvenomous, diurnal, ground-dwelling, and relatively slow-moving animal; it can reach 1.25 m long and 1.1 kg. The large whip snake is a fast, diurnal, nonvenomous, ground-dwelling snake that can exceed 2 m in length and weigh over 2 kg. The eastern Montpellier snake is a subvenomous, aggressive, rear-fanged species notable for its climbing abilities and frequent presence in shrubs and trees; it can reach a length and mass similar to that of the large whip snake (25).

Given these circumstances, we would expect the abundance of glass lizards to decline and snakes to increase during periods of high occupation intensity (i.e., the architectural stage), when economic demands and relative sedentism would push to expand the dietary spectrum to include lower-ranking species. However, EWT’s archaeological record does not fully align with these predictions. The glass lizard appears to have been consistently exploited across all levels, only declining in the more ephemeral postarchitectural and LN stages. It seems to have served as a reliable supplementary food source, regardless of occupation intensity. Similarly, the eastern Montpellier snake does not follow the expected trend, becoming most abundant in the postarchitectural stage. However, unlike the glass lizard, this species’ taphonomic history suggests that much of the increase might be due to animal predation rather than human behavior, with 56% of remains showing evidence of digestion. Last, the large whip snake follows the pattern expected for lower-ranking prey, peaking in the architectural and LN stages and declining in the less intensive postarchitectural stage. We tentatively suggest that these species maintain stable local populations even under sustained human harvest pressure, and likely, constant anthropogenic environmental modification around the site.

To conclude, our study showcases the importance of squamate reptiles in the diet of the early sedentary Natufian communities, as part of the broad-spectrum subsistence strategies characteristic of this period. The first integration of both lizards and snakes into prey-rank models suggests squamate resilience rather than vulnerability to predation pressure, with high- and medium-ranked species consistently targeted despite fluctuations in site occupation intensity. Squamate consumption at large emerges as a sensitive proxy for site occupation intensity and emerging sedentism, tracking shifts in habitation patterns throughout the Natufian sequence. This stands in contrast to pre-Natufian assemblages, which have fewer large-bodied squamates and lower proportions of squamate remains overall, emphasizing the impact of human consumption during the Natufian. Thus, we observed that terminal Pleistocene foragers set in a highly diverse Mediterranean environment exploited squamates regularly at a context of sedentarization and subsistence intensification, and that these animals’ contribution to Natufian diet appears to be remarkable and consistent. Taken together, our results highlight the potential of detailed squamate zooarchaeology to reveal aspects of paleoecology and prehistoric human behavior and the need for better referential frameworks with which to interpret past human–squamate relationships.

## Materials and Methods

The el-Wad Terrace herpetofauna (reptile and amphibian) remains were collected during excavations conducted in 2001–2022 on behalf of the University of Haifa. All excavated sediments underwent wet sieving through 5 mm and 1 mm meshes and were later hand-picked. These remains spanned the site’s entire sequence (15), and the taphonomy of those from the EN architectural stage and the LN stage, have been the object of a former study, which established our method for reconstructing squamate depositional histories (13). The herpetofaunal remains of the pre- and postarchitectural EN stages were not studied prior to the study reported here.

To ensure compatibility, our present methods follow those used in our previous works (13, 26). Our counts primarily drew on the NISP, which are specimens that can be confidently identified to a skeletal element or a part thereof and assigned to a taxon [species, genus, family, or suborder (either lizards or snakes)]. From the specimens assigned to the species level, we also calculated the minimum number of individuals (MNI). We used MNI for the habitat weighting analysis to avoid overrepresenting snakes relative to lizards and anurans, due to the former’s larger number of vertebrae.

Taxonomic identification relied on the comparative collections of the University of Haifa Laboratory of Archaeozoology, the Tel Aviv University Steinhardt Natural History Museum, and the National Natural History Collections at the Hebrew University, Jerusalem. We also relied on Bailon (27, 28), Bailon and Hossini (29), Gleed-Owen (30), Sanchiz (31), Blain et al. (32, 33), Čerňanský et al. (34), Delfino et al. (35), Holman (36), Klembara et al. (37, 38), Villa and Delfino (39), Smith et al. (40), and Szyndlar (41–43).

All identified specimens were examined for bone-surface modifications, following Lev et al.’s (13) experimentally derived procedure and terminology. This was performed under a stereoscopic microscope (Zeiss Discovery V.8) at ×10 to ×80 magnifications and with a high-intensity direct light source. We sought signs of digestion (Type A), trampling and erosion (Types B and E, respectively), digestion altered by trampling and erosion (Type C), bone surface cracking and flaking (Type D and G, respectively), and cutmarks (Type F). Burning was assessed based on bone color, ranging from unburnt to fully calcined. Vertebrae breakage patterns were documented using the protruding parts breakage index (13).

Significantly, various agents are likely to have produced the herpetofaunal assemblages at EWT, including raptor predation, human consumption, and non-predator-related mortality due to entrapment, overwintering, or incidental death. We regard digestion marks as indicators of raptor predation (13). Indices of human exploitation and nonpredatory mortality are more intricate. The former includes cutmarks, the prevalence of large-bodied species, low taxonomic evenness, and intrasite associations with other anthropogenic refuse materials (13, 14, 26), whereas the latter may be characterized by high skeletal completeness, minimal depositional damage (e.g., digestion and anthropogenic alterations), and partial anatomical articulation (44, 45).

Following Szyndlar (41), squamate body size was estimated according to the greatest centrum length (GL) of sufficiently complete trunk vertebrae. As the largest elements in the vertebral column, the size range of trunk vertebrae varies less than that of cervical and caudal vertebrae and is thus particularly suitable for body-size measurements (41).

We reconstructed the site’s paleoenvironments with habitat weightings [or fidelity; (46–49)]. Each species is given a score of 1 that is divided according to its preferred habitats, so that if a given species inhabits more than one habitat, the score is divided accordingly. Following Lev et al. (26, 50), we assigned the identified species proportionally to one or more of the potential habitats using ArcGIS Pro software. The habitats include 28 vegetation units [*SI Appendix,* Fig. S1*A*; revised after Zohary, (51, 52)], and species’ allocations were based on distribution data from modern herpetofauna sightings documented across Israel by the Hebrew University and the Israel Nature and Parks Authority in BioGIS 2020 [Israel Biodiversity Information System (http://www.biogis.huji.ac.il)] and GBIF [Global Biodiversity Information Facility (https://www.gbif.org), *SI Appendix*, *SI References* 43 to 63). The score of habitat types for each species was multiplied by the species MNI and then added to the other species to obtain a picture of the habitats represented in each sample.

To assess taxonomic variations across the site’s Natufian stages, as well as other comparative archaeological and recent assemblages, we applied Simpson’s Index (53), NMDS analysis with Bray–Curtis dissimilarities and permutational multivariate ANOVA (perMANOVA) analyses. NMDS analysis is a distance-based ordination technique that uses rank orders rather than absolute abundances. The statistical analyses were performed in R v. 4.1.2 (54), using the Vegan R-package (55). We compared the EWT taxonomic counts with all available publications from the Levant with detailed taxonomic and taphonomic data: the late Lower Paleolithic Qesem Cave from the Samaria Hills [focusing on two concentrations, >300 ka and ca. 280 to 250 ka; (40)], the Lower Paleolithic sequence of Gesher Benot Ya’aqov (GBY, ca. 780 to 680 ka), the late Middle Paleolithic site of Nahal Mahanayeen Outlet [NMO, ca. 60 ka; (56)], and the Final Natufian site of Eynan [ca. 12 ka; (14)] from the Hula Valley in northeast Israel. From Mount Carmel, our study area, we drew on the Middle Paleolithic assemblages from Layers C–B of Tabun Cave [ca. 160 to 100 ka; (26)] and LN Raqefet Cave [ca. 14 to 12 ka; (50)]. We also analyzed a recent owl pellet assemblage from EWT deposited in the last few decades (13, 26), EWT Locus 25, a nondomestic heap of stones outside the living compound (13), and Raqefet B5, a supposed natural raptor accumulation (50).

To evaluate the foraging value of the main squamate species at EWT, we assessed each taxon against five ranking variables: body size, speed/agility, risk, ecological access, and activity patterns. These variables collectively determine the costs and returns associated with capturing each species.

## Supplementary Material

Appendix 01 (PDF)

## Data Availability

All study data are included in the article and/or *SI Appendix*.
