# Supplementary material for: Three millennia of lizard and snake consumption by Natufian foragers
Source: Proc Natl Acad Sci U S A. 2026 Aug 17;123(35):e2609354123. doi: 10.1073/pnas.2609354123 (PMC13535212; doi:10.1073/pnas.2609354123)
Supplement: Supplementary file 1 [file pnas.2609354123.sapp.pdf]

PNAS

Supporting Information for
Three millennia of lizard and snake consumption by Natufian Foragers

Ma'ayan Lev[1,2]*, Mina Weinstein-Evron[1], Reuven Yeshurun[1]**
[1] School of Archaeology and Maritime Cultures, Zinman Institute of Archaeology, University of Haifa, Haifa 3103301, Israel
[2] Archaeology Stable Isotope Laboratory, Institute for Prehistoric and Protohistoric Archaeology, University of Kiel, 24118, Germany.*

Email:
* Corresponding author. Email: mlev@ufg.uni-kiel.de
** Corresponding author. Email: ryeshuru@research.haifa.ac.il

This PDF file includes:

Supporting text
Figures S1 to S2
Tables S1 to S6
SI References

Supporting Information Text

Section 1. Squamates in past and present human diets

Ethnohistoric and ethnographic research has documented the regular inclusion of squamates in human diets, primarily in tropical or warm desert environments where reptiles are abundant. In Central Africa, the Azande horticulturalists regularly consumed pythons, puff adders, and *Varanus* lizards, but not smaller species (1), and Mbuti hunter-gatherer women collected lizards (2). In tropical South America, groups such as the Nambicuara and Pumé collected and ate various snakes and lizards, including tegu (*Tupinambis teguixin*) and tend lizards (*Ameiva ameiva*), particularly where large game was scarce (3-5). Sometimes, children also caught reptiles, for example, green iguana (*Iguana iguana*) on Colombia's Caribbean Coast (6). In Australia's Western Desert, Aboriginal hunter-gatherers hunted a broad range of squamates, including the centralian blue tongue (*Tiliqua multifasciata*), the great desert skink (*Egernika kintorel*), monitors (*Varanus gouldii* and *V. acanthurus*), pythons (*Aspidites melanocephalus* and *Aspidites ramsayt*), and dragon lizards (*Amphibolurus* sp.). Adults targeted the large-bodied species while children often caught and ate dragon lizards (7-8). In Arabia, Bedouins traditionally hunted the spiny-tailed lizard (*Uromastyx aegyptia*), typically snaring it or digging it out of its burrow (9). Squamates are still consumed in various contemporary contexts, including iguana farming in Central America and python farming in Southeast Asia (10-11).

Despite this rich ethnographic record, only a handful of archaeological attempts to consider squamate consumption can be cited. Monchot et al. (12) drew on intrasite bone distributions and the presence of butchery marks todemonstrate that the spiny-tailed lizard (*Uromastyx aegyptia*) was consumed in 4th–7th century AD Arabia. Dunne et al. (13) cited the high frequency of Nile monitor lizard remains (*Varanus niloticus*) in a Mesolithic/Neolithic camp in Sudan to argue that they were cooked. Shi et al. (14) alluded to the consumption of large-bodied snakes (*Elaphe moellendorffi*, *Ophiophagus hannah*, and *Python bivittatus*) in Neolithic deposits in south China but provided no corroborating evidence. Bochaton et al. (15) compared natural cave and open-air archaeological assemblages to argue for the consumption of *Pholidoscelis* lizards by Amerindians in the Guadeloupe Islands. In a subsequent review, they further claimed that iguanas (*Iguana* spp. and *Cyclura* spp.) are ubiquitous and "were definitely consumed by Indigenous populations" (16:8). However, no supporting evidence was given.

Focusing more narrowly on the late Pleistocene Levant, several researchers have noted the abundance of the European glass lizard (*Pseudopus apodus*) bones in archaeological sites (many of which were burnt) and suggested it might have been consumed (e.g., 17, 18). Recently, we proposed that the European glass lizard and large colubrid snakes were consumed in the Early Natufian site of el-Wad Terrace (19). We based this claim on these remains' association with unambiguous food refuse deposits (i.e., cut and fractured bones of gazelle, hare, game birds, tortoises, and foxes) and the observation of potential cutmarks on six snake vertebrae. Biton et al. (20) performed a similar analysis on the Final Natufian faunal assemblage of Eynan and argued for the consumption of large-bodied colubrid snakes. In subsequent studies, we observed that, compared to earlier assemblages (Tabun Cave) and contemporary collections, Natufian herpetofaunal communities feature more large-bodied individuals and are dominated by fewer taxa (21,22). This difference appears to be primarily anthropogenic, attributable to regular and purposeful Natufian squamate consumption.

Section 2. The archaeological case-study

El-Wad is part of the Nahal Me'arot cave complex, a UNESCO World Heritage site located at the foothills of the western escarpment of Mount Carmel (Israel), ca. 45 m above sea level, at the outlet of a seasonally-flowing channel to the Mediterranean Coastal Plain. The present-day climate is Mediterranean with hot, dry summers and cool, rainy winters (23). Precipitation,

temperature, and vegetation vary with elevation and proximity to the coast. Annual rainfall ranges from 564 to 692 mm and mean temperatures from 18.1 to 20.1 °C (data from 'Ein Carmel and the University of Haifa meteorological stations). The vegetation is primarily East Mediterranean. The lower slopes (< 300 m AMSL) are characterized by an evergreen park forest dominated by *Ceratonia siliqua* and *Pistacia lentiscus*, while higher elevations in the mountain's center and east feature dense evergreen woodlands with *Quercus calliprinos and Pistacia palaestina*. South-facing slopes support relatively open grass-rich habitats, whereas north-facing slopes resemble the dense evergreen woodlands. Additionally, nearby habitats include Q*. ithaburensis* park forest in the southeastern parts of the mountain and the adjacent Menashe Plateau, rich bank vegetation in alluvial valleys, and herbaceous communities on the Coastal Plain's *kurkar* ridges and sand dunes (24–26; for a more detailed description, see 22; *SI Appendix* Fig. S1).

Pollen and anthracological remains from the Natufian deposits in el-Wad confirmed that the region featured a broadly similar Mediterranean maquis in the latest Pleistocene, albeit with some notable shifts in the relative frequencies of evergreen oaks, deciduous oaks, almond, and tamarisk along the sequence (27–29). Isotopic studies ($\Delta^{13}C$) of directly-dated charred almond remains indicate high rainfall throughout most of the Early Natufian sequence, followed by a slight decrease towards the end and crossing over to the Late Natufian. Similarly, our previous herpetofaunal-based paleoclimatic reconstruction showed that, compared to earlier (Middle Paleolithic) deposits at the Mount Carmel Caves and recent samples (22), the woodlands were most expanded during the Early Natufian.

The present study draws on the recent excavations of the northeastern part of el-Wad Terrace (henceforth EWT). This excavation revealed a rich sequence of Natufian habitations of varying intensity (30, 31). The Early Natufian (EN, 15,000–13,700 cal. BP) at el-Wad consists of three distinct stages spanning 18 levels. We dub the first stage the *pre-architectural stage* (Pre-arch; Phase W-8, Levels XVII–XIII). It includes several small stone features but no major architectural construction. Next is the *architectural stage* (Arch; Phases W-6/W-7, Levels IX–IV); it features stone constructions, most notably dwelling structure remains (stone walls and abutting floors), stone heaps, artifacts, and burnt materials enclosed in a curvilinear wall (30), along with strikingly high densities of faunal and charred wood remains. Together, these observations suggest that a peak in habitation intensity and sedentism was reached at this stage (29, 31). The final EN stage is the *post-architectural stage* (Post-arch; Phase W-5, Levels III–I, and Phases W-4 and W-3); it consists of well-preserved late EN layers with a few stone features but no architectural remains, perhaps underscoring a return to a less permanent mode of site occupation (30). El-Wad's EN sequence was capped by a Late Natufian layer (LN, 13,700–12,000 cal. BP; Phase W-0 and Unit 1-2). It includes a cemetery and other deposits, but no architecture. Nevertheless, the broad-spectrum foraging patterns suggest that significant domestic activity took place during this stage, albeit probably reduced (32). According to Bayesian modeling of the available radiocarbon dates, we estimate that the EN sequence was 700 years long at most and possibly as brief as 250 years (31).

The history of paleozoological research at Natufian el-Wad is exceptionally long, commencing with Bate's (33) quantitative and systematic account of the fauna from the first campaign in 1929–1933, and growing in methodical and theoretical sophistication ever since (34, and references therein). The Natufian hunting economy is notable for the abundance of mountain gazelle (*Gazella gazella*), the smallest ungulate in the region, and numerous small-bodied species (32; 35–39), including mole-rats (*Spalax* sp.), hare, *Lepus capensis*, small carnivorans, aquatic birds, and tortoises. This Natufian broad-spectrum subsistence strategy was attributed to resource intensification, which was driven by initial sedentism (32,40).

While these economic patterns generally persisted throughout the Natufian sequence, some variability was noted, coinciding with other proxies of shifting site use. Thus, the pre-architectural stage manifests a diversified, albeit moderately intense, economy. In the

subsequent architectural stage, habitation intensity at the site peaked, whereas in the post-architectural stage, more specialized or seasonal hunting patterns were practiced, and the site was more frequently abandoned (31). How squamates fit into this occupational sequence is yet to be systematically examined. Notwithstanding its long history of research, el-Wad's squamate remains were not studied until recently (19, 22). Addressing this gap is the aim of the present paper.

Section 3. Extended taphonomic analysis

Digestion marks were noted in all EWT levels (*SI Appendix* Fig S2A; *SI Appendix* Table S3). They were quite frequent in the pre-architectural and architectural stages (10%–34% and 24%–34% of identified squamate bones, respectively), increased in frequency in the post-architectural stage (35%–62%), and decreased in the LN stage (14%–15%). Notably, the prevalence of digestion modifications varies across the three main species at the site: The percentages are low for the European glass lizard and the large whip snake (13% and 9%, respectively) but high for the eastern Montpellier snake (38%).

Bone burning is very common (*SI Appendix* Fig S2B; *SI Appendix* Table S3), including most identified bones in the pre-architectural and architectural stages (59%–81% and 55%–70%, respectively), and approximately half or less in the post-architectural and LN stages (36%–49% and 44%–54% respectively). Concerning breakage (*SI Appendix* Fig S2C; *SI Appendix* Table S3), the pre-architectural samples have the lowest indices (0.49–0.57), followed by a significant increase in the architectural stage (0.72–0.77), a decrease in the post-architectural stage (0.49–0.58), and a mild increase in the LN (0.64–0.68).

Bone surface cracking and flaking were common in all EWT levels (*SI Appendix* Fig S2D,E; *SI Appendix* Table S3). Nevertheless, some variation was noted: Cracking and flaking seem to have been more intense in the pre-architectural and post-architectural stages than in the architectural and LN stages. Notably, these modifications are related to various organic and inorganic processes and are often linked to digestion and weathering processes (41). However, we found no correlation between the relative frequency of digestion marks to cracking or flaking along the sequence (Spearman's R= 0.16, p=0.36, and R= 0.03, p=0.53, respectively), suggesting that these modifications were more likely produced by lengthy exposure to the elements.

Figures

Fig. S1. A) Vegetation map for habitat weighting analysis B) Vegetation map of Mount Carmel. Revised from Zohary (1962, 1982).

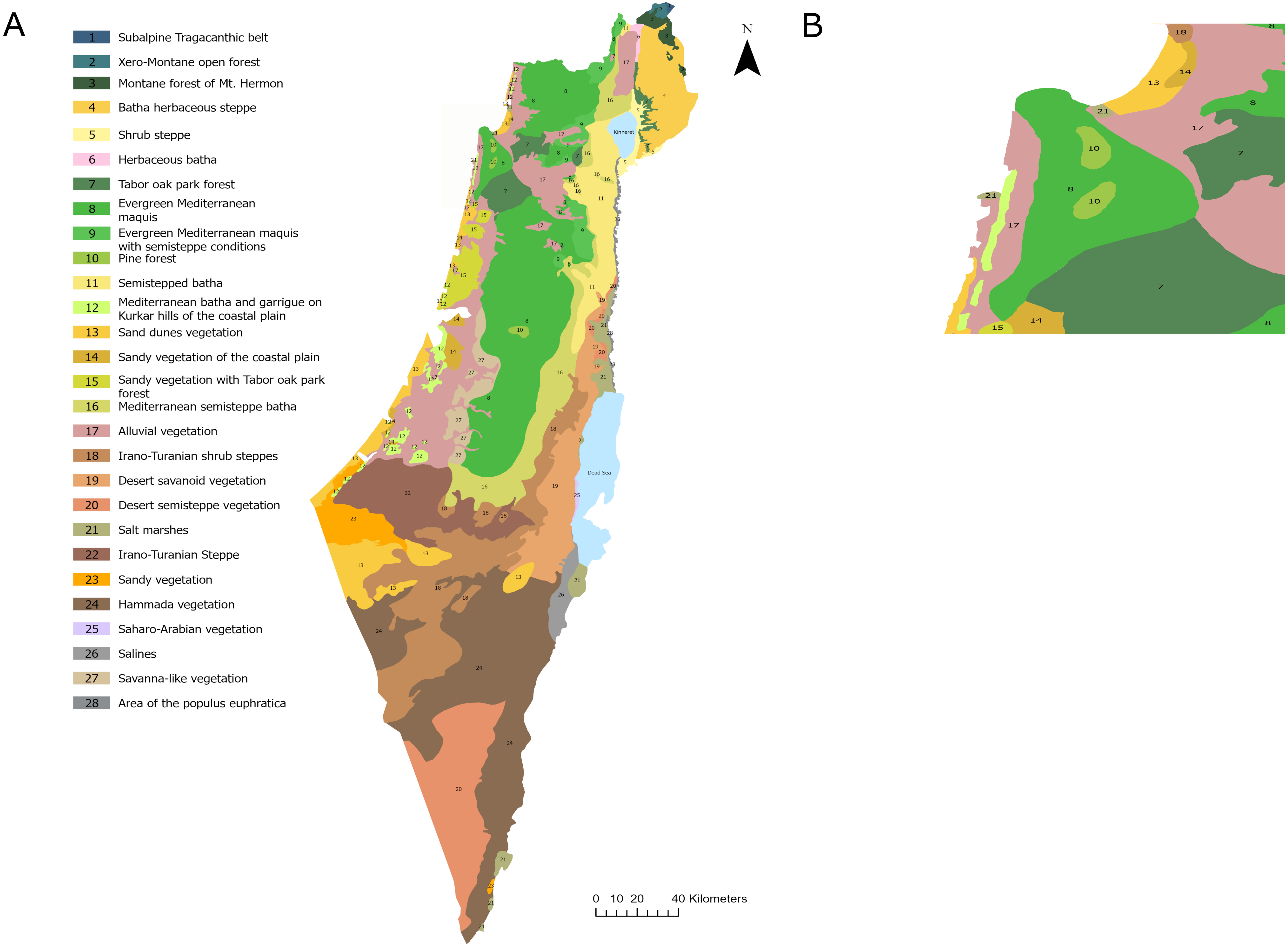
A
B
1 Subalpine Tragacanthic belt
2 Xero-Montane open forest
3 Montane forest of Mt. Hermon
4 Batha herbaceous steppe
5 Shrub steppe
6 Herbaceous batha
7 Tabor oak park forest
8 Evergreen Mediterranean maquis
9 Evergreen Mediterranean maquis with semisteppe conditions
10 Pine forest
11 Semistepped batha
12 Mediterranean batha and garrigue on Kurkar hills of the coastal plain
13 Sand dunes vegetation
14 Sandy vegetation of the coastal plain
15 Sandy vegetation with Tabor oak park forest
16 Mediterranean semisteppe batha
17 Alluvial vegetation
18 Irano-Turanian shrub steppes
19 Desert savanoid vegetation
20 Desert semisteppe vegetation
21 Salt marshes
22 Irano-Turanian Stepppe
23 Sandy vegetation
24 Hammada vegetation
25 Saharo-Arabian vegetation
26 Salines
27 Savanna-like vegetation
28 Area of the populus euphratica
N
Kinneret
Dead Sea
0 10 20 40 Kilometers

Fig. S2. Frequencies of taphonomic features along the EWT stratigraphic sequence. (A) proportion of digested bones (Types A and C); (B) proportion of burnt bones; (C) breakage index: protruding part index, measuring bone fragmentation; (D) cracking intensity; (E) flaking intensity.

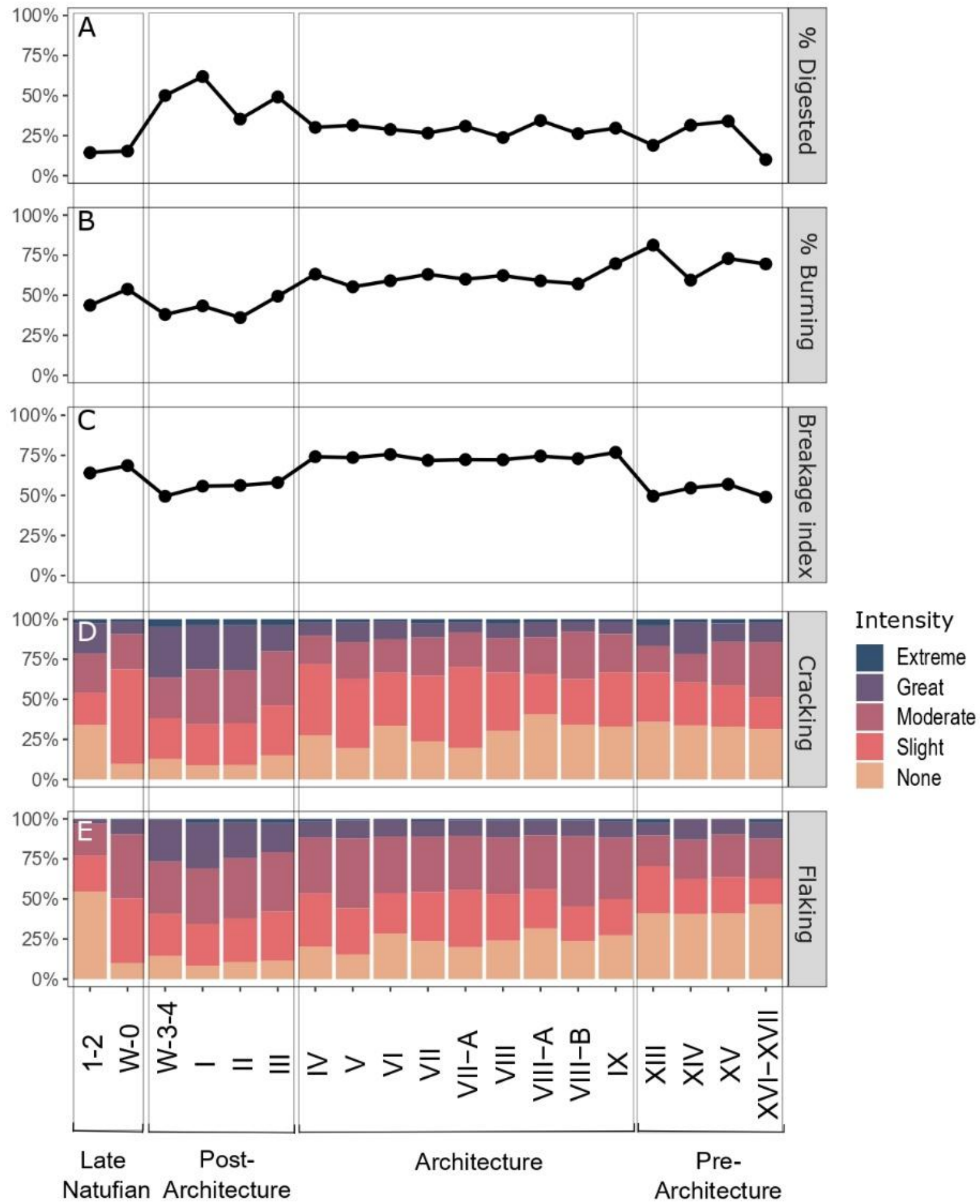

Tables

Table S1. NISP counts and proportional abundances of squamates and "edible squamates" in the EWT sequence, based on the specimens from the 5 mm mesh only.

	UNIT 1-2	W-0	W-3-4	I	II	III	IV	V	VI	VII	VII-A	VIII	VIII-A	VIII-B	IX	XIII	XIV	XV	XVI-XVII
Small mammal	71	74	43	39	41	33	98	75	70	95	79	77	92	41	52	27	18	66	24
Small ungulate	525	470	190	227	242	242	401	471	320	473	294	379	489	215	224	58	80	228	114
Medium ungulate	9	7	11	5	16	12	17	21	12	13	11	15	13	9	6	7	3	17	5
Large ungulate	3	5		1				1		1	2		1	3	1		1	2	0
Bird	6	4	8	4	9	5	1	10	3	6	10	13	13	10	6	4	3	18	2
Tortoise	199	109	42	45	51	53	118	148	77	128	111	130	123	68	70	26	31	84	42
Lizard & snake	478	479	144	125	150	117	281	331	216	385	274	290	354	183	243	68	82	280	88
Total	1291	1148	438	446	509	462	916	1057	698	1101	781	904	1085	529	602	190	218	695	275
Edible squamates	153	232	16	10	26	18	103	128	93	174	103	113	121	77	84	21	14	56	18
Squamates/total NISP	0.370256	0.417247	0.328767	0.280269	0.294695	0.253247	0.306769	0.31315	0.309456	0.349682	0.350832	0.320796	0.326267	0.345936	0.403654	0.357895	0.376147	0.402878	0.32
Edible squamates/total NISP	0.118513	0.202091	0.03653	0.022422	0.051081	0.038961	0.112445	0.121097	0.133238	0.158038	0.131882	0.125	0.111521	0.145558	0.139535	0.110526	0.06422	0.080576	0.065455

Table S2. Taxonomic composition of the EWT Natufian herpetofauna assemblages.

	LN		Post- architectural				Architectural									Pre- architectural			
	1-2	W-0	W-3-4	I	II	III	IV	V	VI	VII	VII-A	VIII	VIII-A	VIII-B	IX	XIII	XIV	XV	XVI–XVII
**Amphibia**																			
Pelobates cf. *syriacus*	1	1	1					1			1							2	
Bufotes cf. *sitibundus*	3	6				1		2		1	1						1	3	
Pelophylax bedriagae									1										
Anura indet.	7	8						1	3	1	2	3		1	2			1	
Total Amphibia	11	15	1			1		4	4	2	4	3		1	2		1	6	
**Squamata**																			
**Lizard**																			
Stellagama cf. *stellio*	11	21	2	1	4	3	8	12	9	21	4	11	20	7	9	3	3	6	2
Chamaeleo cf. *chamaeleon*		1	1		1	1					1		1						
Eumeces schneideri	3	1					2		1		1								
Pseudopus apodus	55	81	24	32	27	24	81	77	51	106	74	71	90	40	68	14	24	47	13
Agamidae Indet.				2	1													2	
Lacertidae Indet.		1							1			1	1						
Scincidae indet.	3	2	1		1	2	2	3	1	2		1	4	1			1		1
Lizard indet.	20	20					15	11	15	15	8	9	12	5	9				
Total lizards	92	127	28	35	34	30	108	103	78	144	88	93	128	53	86	17	28	55	16
**Snake**																			
Eryx cf. *jaculus*	3	1		2	3	2	1	2	1	3	2	1	2		2				1
Malpolon insignitus	74	60	26	39	37	32	40	51	37	51	37	30	39	25	24	7	7	24	12
Psammophis schokari	1				1	1											1		2
Dolichophis jugularis	90	104	20	15	28	29	46	59	56	102	47	67	77	40	44	12	13	36	16
Elaphe cf. *sauromates*			1		1					1									

Hemorrhois nummifer	2				1		3	1	1	2	1	2	1		2		3	1	2
Platyceps collaris									1										
Telescopus cf. *fallax*	2									1									
Daboia cf. *palaestinae*	3	11	4	4	3	1	2	4		6	3	3	1	1	2		1	4	5
Colubridae indet.	231	255	74	50	67	45	179	193	153	234	154	191	282	143	195	40	35	138	45
Colubridae-Colubrinae	23	22	4		3	6	11	16	12	18	11	10	16	4	11		2	13	1
Colubridae-Psamophiininae	9	7	2	1		3	5	8	6	6	9	7	15	2	9	1	4	4	6
Viperidae indet.	2	10					10	4	5	10	5	4	7	1	1		1	4	5
Serpentes indet.	168	190	6	11	11	7	75	108	78	100	89	119	94	42	84	3	5	28	12
Total snakes	608	648	137	122	155	126	362	442	345	524	353	430	527	257	373	63	71	248	102

Table S3. Taphonomic table of the EWT levels divided by stages.

	LN		Post- architecture				Architecture									Pre- architecture			
	1-2	W-0	W-3/4	I	II	III	IV	V	VI	VII	VII-A	VIII	VIII-A	VIII-B	IX	XIII	XIV	XV	XVI-XVII
Total NISP	714	802	166	157	189	158	480	554	432	681	450	530	663	312	462	80	101	310	118
Type A																			
n	6	2							1	1	3	2	3		3		1		
%	0. 8	0. 2	0	0	0	0	0	0	0. 2	0. 1	0. 7	0. 4	0. 5	0. 0	0. 6	0. 0	1.00	0.000	0.000
slight	1	0																	
moderate	0	0																	
great	0	0							1		2		1		1				
extreme	5	2								1	1	2	2		2		1		
Type B																			
n	429	525	126	107	144	112	247	289	218	360	228	278	317	178	241	59	68	200	75
%	60	65	76	68	76	71	51	52	50	53	51	52	48	57	52	74	67	65	64
slight	71	119	13	9	18	3	45	49	29	65	53	49	43	27	42	12	2	16	3
moderate	200	177	41	35	47	41	111	112	101	141	105	136	123	62	107	25	35	82	36
great	144	202	61	49	61	60	71	112	78	137	61	84	140	84	86	20	26	85	31
extreme	14	27	11	12	17	7	20	15	10	17	9	9	10	5	6	2	4	12	4
index	0.44	0.52	0.60	0.64	0.63	0.65	0.57	0.58	0.58	0.57	0.53	0.55	0.59	0.59	0.56	0.55	0.61	0.60	0.61
Type C																			
n	45	106	25	32	23	28	97	123	73	131	100	73	140	54	83	5	10	47	4
%	10	13	15	20	12	18	20	22	17	19	22	14	21	17	18	6	10	15	3
slight	14	4	1	3	0	0	5	11	7	14	14	5	17	9	15	0	0	5	1
moderate	27	65	15	16	15	16	60	68	35	70	52	48	68	21	50	3	7	26	2
great	4	34	8	12	8	10	27	39	31	42	31	18	50	18	17	2	3	15	1
extreme	0	3	1	1		2	5	5		5	3	2	5	6	1			1	
Type D																			
n	431	676	144	143	171	136	330	396	270	474	325	322	374	197	298	50	67	205	72
%	60	84	87	91	90	86	69	71	63	70	72	61	56	63	65	63	66	66	61

none	221	72	21	14	17	24	125	96	136	148	80	140	257	102	147	28	34	101	33
slight	131	442	42	40	49	50	202	213	134	254	204	168	158	85	150	24	27	78	21
moderate	160	164	42	54	62	54	82	113	85	149	87	100	145	89	107	13	18	84	36
great	124	59	52	43	53	26	37	60	46	56	26	41	59	19	35	10	20	35	13
extreme	16	11	8	6	7	6	9	10	5	15	8	13	12	4	6	3	2	8	2
index	0.34	0.33	0.48	0.48	0.48	0.41	0.28	0.33	0.28	0.31	0.30	0.29	0.27	0.28	0.28	0.29	0.32	0.31	0.36
Type E																			
n	474	631	151	138	166	139	344	411	291	491	329	352	461	233	324	65	77	243	78
slight	85	123	14	12	18	3	50	60	36	79	67	54	60	36	57	12	2	21	4
moderate	227	242	56	52	62	57	171	180	136	211	157	184	194	84	157	29	42	108	38
great	148	236	69	61	69	70	98	151	109	179	93	103	192	102	103	22	29	101	32
extreme	14	30	12	13	17	9	25	20	10	22	12	11	15	11	7	2	4	13	4
erosion index	0.44	0.52	0.60	0.59	0.57	0.59	0.46	0.51	0.45	0.48	0.47	0.44	0.47	0.49	0.44	0.49	0.50	0.50	0.52
Type G																			
n	295	670	141	142	168	141	363	416	290	475	326	351	430	229	323	46	60	181	56
%	41	84	85	90	89	89	76	75	67	70	72	66	65	73	70	58	59	58	47
none	356	75	24	13	20	18	92	75	115	148	81	111	199	71	121	32	41	126	49
slight	144	300	43	40	51	49	151	142	102	191	145	134	153	65	101	23	22	69	17
moderate	132	298	54	54	71	59	159	214	143	214	138	163	213	132	170	15	25	82	26
great	19	67	43	44	42	29	46	55	42	62	39	49	57	29	46	6	13	30	11
extreme	0	5	1	4	4	4	7	5	3	8	4	5	7	3	6	2			2
index	0.18	0.50	0.43	0.48	0.45	0.42	0.35	0.38	0.32	0.34	0.34	0.34	0.31	0.36	0.34	0.25	0.27	0.26	0.29
burning																			
n	312	431	63	68	68	78	303	306	255	429	270	330	391	178	322	65	60	226	82
%	44	54	38	43	36	49	63	55	59	63	60	62	59	57	70	81	59	73	69
protruding index																			
n	358	502	89	83	92	83	308	329	275	429	253	312	445	208	306	45	50	155	57
0	8	5	14	6	9	2	8	6	1	10	5	6	7	7	2	4	5	10	4

1	37	36	12	13	13	10	14	21	13	29	22	23	24	6	17	8	7	18	11
2	33	30	7	7	13	10	19	22	15	22	11	21	24	11	13	2	4	20	4
3	56	67	18	15	7	16	31	22	30	36	20	32	28	29	27	10	10	19	13
4	33	62	5	6	7	7	29	31	21	59	24	17	48	15	24	8	1	12	8
5	47	101	11	10	8	13	35	55	47	53	43	47	75	29	43	3	10	31	7
6	51	58	3	6	13	11	48	46	40	74	36	56	84	40	56	4		19	1
7	93	143	19	20	22	14	124	126	108	146	92	110	155	71	124	6	13	26	9
protruding index	0.64	0.68	0.49	0.55	0.56	0.58	0.74	0.73	0.75	0.72	0.72	0.72	0.74	0.73	0.77	0.49	0.54	0.57	0.49
completeness																			
1	52	102	4	3	6	3	72	72	57	93	66	63	123	41	80	3	7	10	5
2	197	251	42	47	48	65	162	184	150	229	152	181	231	118	167	26	24	90	23
3	208	335	64	67	88	44	179	196	164	259	140	176	229	114	152	23	35	132	32
4	199	72	56	40	47	48	58	65	44	69	69	60	73	35	57	26	36	79	35
index	0.71	0.62	0.76	0.73	0.73	0.71	0.62	0.62	0.62	0.62	0.62	0.62	0.60	0.62	0.60	0.73	0.75	0.73	0.76
Digestion																			
none	328	586	27	21	44	29	237	272	200	368	231	266	282	152	211	30	24	99	36
slight	12	17	3			1	4	9	4	10	5	7	14	7	3	1		2	
moderate	1	0	0									1	1						
great	26	43	8	13	15	6	40	50	30	59	47	25	54	16	41	2	3	19	3
extreme	16	46	16	21	9	21	58	66	47	64	51	50	79	31	45	4	8	30	1
total	55	106	27	34	24	28	102	125	81	133	103	83	148	54	89	7	11	51	4
% in measurable bones	14	15	50	62	35	49	30	31	29	27	31	24	34	26	30	19	31	34	10
% of total NISP	8	13	16	22	13	18	21	23	19	20	23	16	22	17	19	9	11	16	3
Total count of cut marks	1	6	2	1	2		5	6	1	12	7	2		2	6	4	2	6	7
Count of cutmarks in the sample	1	2	2	1	2		4	6	1	4	6	2		2	5	3		5	4
% Cut marks in the sample	0.14	0.25	1.20	0.64	1.06	0.00	0.83	1.08	0.23	0.59	1.33	0.38	0.00	0.64	1.08	3.75	0.000	1.61	3.39

Table S4. List of all specimens with identified cutmarks (Type F). EN, Early Natufian, where phase W-8 is the pre-architectural stage, Phases W-7 to W-6 are the architectural stage, Phases W-5 to W-3 are the post-architectural stage, and LN is the Late Natufian. For other stratigraphic details, see Weinstein-Evron et al. (30) and Yeshurun et al. (31).

Catalog number	Unit	Period	Phase	Level	Species
24519	UNIT2	EN	W-1		*Malpolon insignitus*
23883	UNIT2	EN	W-4		*Dolichopis jugularis*
24274	UNIT2	EN	W-4		*Dolichopis jugularis*
24036	UNIT2	EN	W-5	I	*Dolichopis jugularis*
23873	UNIT2	EN	W-5	II	*Dolichopis jugularis*
24143	UNIT2	EN	W-5	II	*Dolichopis jugularis*
2517	UNIT2	EN	W-6	IV	Colubridae indet.
2518	UNIT2	EN	W-6	IV	Colubridae-colubrinae indet.
3192	UNIT2	EN	W-6	IV	*Malpolon insignitus*
7378	UNIT2	EN	W-6	IV	*Malpolon insignitus*
24520	UNIT2	EN	W-6	IV	*Dolichopis jugularis*
1255	UNIT2	EN	W-6	under L20	*Dolichopis jugularis*
2213	UNIT2	EN	W-6	V	*Malpolon insignitus*
2665	UNIT2	EN	W-6	V	*Dolichopis jugularis*
2697	UNIT2	EN	W-6	V	*Malpolon insignitus*
3261	UNIT2	EN	W-6	V	*Dolichopis jugularis*
4737	UNIT2	EN	W-6	V	*Dolichopis jugularis*
7638	UNIT2	EN	W-6	V	*Dolichopis jugularis*
4140	UNIT2	EN	W-6	VI	*Dolichopis jugularis*
6734	UNIT2	EN	W-6	VII	*Dolichopis jugularis*
7785	UNIT2	EN	W-6	VII	*Dolichopis jugularis*
7792	UNIT2	EN	W-6	VII	*Dolichopis jugularis*
22070	UNIT2	EN	W-6	VII	Colubridae indet.

24522	UNIT2	EN	W-6	VII	*Dolichopis jugularis*
24523	UNIT2	EN	W-6	VII	*Dolichopis jugularis*
24524	UNIT2	EN	W-6	VII	*Malpolon insignitus*
24525	UNIT2	EN	W-6	VII	*Malpolon insignitus*
24527	UNIT2	EN	W-6	VII	*Dolichopis jugularis*
24533	UNIT2	EN	W-6	VII	*Malpolon insignitus*
24534	UNIT2	EN	W-6	VII	*Malpolon insignitus*
24539	UNIT2	EN	W-6	VII	Colubridae indet.
3050	UNIT2	EN	W-6	VII-A	*Dolichopis jugularis*
7930	UNIT2	EN	W-6	VII-A	*Malpolon insignitus*
7981	UNIT2	EN	W-6	VII-A	*Dolichopis jugularis*
7982	UNIT2	EN	W-6	VII-A	*Malpolon insignitus*
8019	UNIT2	EN	W-6	VII-A	*Malpolon insignitus*
8059	UNIT2	EN	W-6	VII-A	*Dolichopis jugularis*
24535	UNIT2	EN	W-6	VII-A	*Malpolon insignitus*
3751	UNIT2	EN	W-7	IX	*Dolichopis jugularis*
7320	UNIT2	EN	W-7	IX	*Dolichopis jugularis*
8309	UNIT2	EN	W-7	IX	*Dolichopis jugularis*
8821	UNIT2	EN	W-7	IX	*Dolichopis jugularis*
22260	UNIT2	EN	W-7	IX	Colubridae indet.
24544	UNIT2	EN	W-7	IX	Colubridae indet.
711	UNIT2	EN	W-7	stone-poor layer	Colubridae indet.
21249	UNIT2	EN	W-7	stone-poor layer	Serpentes indet.
6366	UNIT2	EN	W-7	VIII	*Dolichopis jugularis*
6370	UNIT2	EN	W-7	VIII	*Malpolon insignitus*
1330	UNIT2	EN	W-7	VIII-B	Colubridae indet.
1562	UNIT2	EN	W-7	VIII-B	*Malpolon insignitus*
23226	UNIT2	EN	W-8	XIII	Colubridae indet.
23322	UNIT2	EN	W-8	XIII	Colubridae indet.
23706	UNIT2	EN	W-8	XIII	*Dolichopis jugularis*

24582	UNIT2	EN	W-8	XIII	Colubridae indet.
24538	UNIT2	EN	W-8	XIV	*Dolichopis jugularis*
24542	UNIT2	EN	W-8	XIV	*Malpolon insignitus*
23351	UNIT2	EN	W-8	XV	*Dolichopis jugularis*
23381	UNIT2	EN	W-8	XV	*Dolichopis jugularis*
23477	UNIT2	EN	W-8	XV	Colubridae indet.
23563	UNIT2	EN	W-8	XV	*Malpolon insignitus*
23565	UNIT2	EN	W-8	XV	*Dolichopis jugularis*
24531	UNIT2	EN	W-8	XV	Colubridae indet.
23392	UNIT2	EN	W-8	XVI	*Dolichopis jugularis*
23394	UNIT2	EN	W-8	XVI	Colubridae indet.
23437	UNIT2	EN	W-8	XVI	Colubridae indet.
23614	UNIT2	EN	W-8	XVI	Colubridae-colubrinae indet.
24515	UNIT2	EN	W-8	XVI	*Malpolon insignitus*
24541	UNIT2	EN	W-8	XVII	*Malpolon insignitus*
24543	UNIT2	EN	W-8	XVII	Colubridae indet.
1499	UNIT2	EN		stone-poor layer	*Dolichopis jugularis*
1759	UNIT2	EN		stone-poor layer	*Dolichopis jugularis*
24517	UNIT2	EN			Colubridae indet.
24521	UNIT2	EN			Colubridae indet.
24580	UNIT2	EN			*Dolichopis jugularis*
11395	UNIT2	LN	W-0	W-0	Colubridae indet.
11502	UNIT2	LN	W-0	W-0	*Malpolon insignitus*
20255	UNIT1-2	LN	W-0	W-0	Colubridae indet.
24516	UNIT2	LN	W-0		*Dolichopis jugularis*
24518	UNIT2	LN	W-0		*Malpolon insignitus*
24532	UNIT2	LN	W-0		*Dolichopis jugularis*
24526	UNIT2	LN	W-0 - W-2		Colubridae indet.
24513	UNIT1	LN			*Dolichopis jugularis*

Table S5. Habitat weighting for the different levels, habitat names follow.

Phase	Layer	1	2	3	4	5	6	7	8	9	10	11	12	13	14	15	16	17	18	19	20	21	22	23	24	25	26	27	28	29
LN	1_2	0.03	0.14	0.43	1.26	0.11	0.17	0.81	3.68	0.71	0.29	0.65	0.26	1.01	0.29	0.60	0.81	2.31	0.37	0.13	0.12	0.08	0.25	0.07	0.27	0.07	1.02	0.03	0.00	0.01
	W-0	0.02	0.12	0.34	1.11	0.07	0.14	1.04	4.32	0.74	0.31	0.58	0.31	1.18	0.38	0.67	0.68	2.42	0.31	0.07	0.05	0.04	0.23	0.06	0.13	0.00	1.54	0.03	0.00	0.01
Post-arch	W-3-4	0.01	0.06	0.21	0.63	0.06	0.10	0.51	2.04	0.25	0.17	0.31	0.16	0.77	0.15	0.25	0.43	1.26	0.21	0.07	0.06	0.07	0.18	0.06	0.19	0.06	0.62	0.02	0.00	0.00
	I	0.00	0.05	0.15	0.51	0.03	0.06	0.34	1.31	0.16	0.10	0.25	0.14	0.42	0.12	0.21	0.31	0.97	0.09	0.02	0.01	0.03	0.14	0.05	0.02	0.00	0.46	0.02	0.00	0.00
	II	0.01	0.08	0.25	0.75	0.07	0.11	0.56	2.53	0.28	0.20	0.40	0.21	0.96	0.23	0.30	0.64	1.51	0.30	0.07	0.07	0.07	0.21	0.07	0.21	0.06	0.81	0.03	0.00	0.00
	III	0.01	0.07	0.21	0.70	0.05	0.10	0.64	2.46	0.40	0.19	0.37	0.20	1.04	0.24	0.34	0.54	1.52	0.26	0.08	0.07	0.07	0.18	0.07	0.22	0.01	0.94	0.03	0.00	0.00
Arch	IV	0.01	0.08	0.26	0.67	0.06	0.12	0.84	2.81	0.32	0.27	0.44	0.19	0.76	0.20	0.28	0.58	1.60	0.13	0.04	0.03	0.03	0.18	0.05	0.04	0.06	1.00	0.03	0.00	0.00
	V	0.02	0.09	0.31	1.00	0.07	0.13	0.83	3.65	0.57	0.24	0.41	0.27	0.89	0.27	0.57	0.73	2.04	0.23	0.04	0.02	0.03	0.21	0.06	0.09	0.06	1.11	0.03	0.00	0.01
	VI	0.01	0.11	0.27	1.05	0.07	0.11	0.66	2.32	0.29	0.22	0.41	0.17	0.50	0.19	0.33	0.44	1.40	0.13	0.07	0.03	0.03	0.17	0.06	0.04	0.06	0.77	0.02	0.00	0.01
	VII	0.04	0.09	0.41	0.86	0.08	0.17	1.12	4.39	0.63	0.31	0.48	0.27	1.10	0.28	0.43	0.64	2.23	0.23	0.06	0.02	0.03	0.23	0.06	0.08	0.06	1.42	0.03	0.00	0.01
	VII-A	0.01	0.09	0.29	1.01	0.07	0.14	0.89	3.35	0.56	0.30	0.53	0.27	1.00	0.32	0.58	0.60	2.05	0.19	0.06	0.04	0.04	0.20	0.06	0.08	0.06	1.21	0.03	0.00	0.00
	VIII	0.01	0.09	0.29	0.72	0.07	0.12	0.81	3.25	0.34	0.24	0.39	0.22	0.81	0.22	0.31	0.47	1.73	0.21	0.03	0.01	0.03	0.20	0.06	0.05	0.06	1.03	0.03	0.00	0.00
	VIII-A	0.02	0.11	0.35	0.85	0.08	0.13	0.98	4.05	0.40	0.30	0.49	0.28	1.09	0.31	0.38	0.56	2.11	0.29	0.03	0.02	0.03	0.23	0.06	0.07	0.06	1.38	0.03	0.00	0.00
	VIII-B	0.01	0.06	0.19	0.52	0.04	0.07	0.57	2.20	0.23	0.17	0.24	0.14	0.56	0.15	0.18	0.33	1.10	0.13	0.01	0.01	0.02	0.12	0.03	0.03	0.00	0.75	0.02	0.00	0.00
	IX	0.01	0.07	0.30	0.69	0.06	0.14	1.04	3.52	0.38	0.29	0.39	0.23	0.97	0.24	0.33	0.47	1.86	0.15	0.03	0.01	0.03	0.18	0.06	0.04	0.06	1.25	0.03	0.00	0.00
Pre-arch	XIII	0.00	0.05	0.13	0.37	0.02	0.06	0.50	1.68	0.20	0.14	0.18	0.11	0.40	0.11	0.11	0.21	0.77	0.07	0.01	0.01	0.01	0.08	0.03	0.02	0.00	0.64	0.01	0.00	0.00
	XIV	0.01	0.06	0.22	0.64	0.06	0.11	0.62	2.56	0.44	0.20	0.27	0.14	0.82	0.16	0.23	0.43	1.28	0.21	0.07	0.06	0.06	0.15	0.04	0.22	0.07	0.76	0.02	0.00	0.00
	XV	0.02	0.08	0.29	0.93	0.07	0.12	0.79	3.57	0.57	0.24	0.35	0.23	0.81	0.25	0.48	0.49	1.80	0.21	0.03	0.02	0.02	0.17	0.03	0.09	0.06	1.05	0.02	0.00	0.01
	XVI-	0.01	0.06	0.21	0.63	0.06	0.10	0.51	2.04	0.25	0.17	0.31	0.16	0.77	0.15	0.25	0.43	1.26	0.21	0.07	0.06	0.07	0.18	0.06	0.19	0.06	0.62	0.02	0.00	0.00

	X<br>VII																													

1	Subalpine Tragacanthic belt
2	Xero-Montane open forest
3	Montane forest of Mt. Hermon
4	Batha herbaceous steppe
5	Shrub steppe
6	Herbaceous batha
7	Tabor oak park forest
8	Evergreen Mediterranean maquis
9	Evergreen Mediterranean maquis with semisteppe conditions
10	Pine forest
11	Semistepped batha
12	Mediterranean batha and garrigue on Kurkar hills of the coastal plain
13	Sand dunes vegetation
14	Sandy vegetation of the coastal plain
15	Sandy vegetation with Tabor oak park forest
16	Mediterranean semisteppe batha
17	Alluvial vegetation
18	Irano-Turanian shrub steppes
19	Desert savanoid vegetation
20	Desert semisteppe vegetation
21	Salt marshes
22	Irano-Turanian Steppe
23	Sandy vegetation
24	Hammada vegetation
25	Salines
26	Savanna-like vegetation
27	Area of the populus euphratica

28	Dead Sea
29	Kinneret

Table S6 Prey-rank evaluation of the main squamate species identified at EWT based on five ranking variables (data from 42)

Species	Speed/agility	Body size	Risk	Ecological access	Activity pattern	Overall rank
European glass lizard (*Pseudopus apodus*)	Slow	Large (up to 1250mm, 3.1-1100g)	Low (non-venomous)	High (local, abundant)	Diurnal	High
Large whip snake (*Dolichophis jugularis*)	Fast	Large (330-2510mm, 4.6-2400g)	Medium (non-venomous, strong bite)	High (local, abundant)	Diurnal	Medium-High
Eastern Montpellier snake (*Malpolon insignitus*)	Fast	Large (260-2083mm, 4-1800g)	Medium-High (mildly venomous, aggressive)	High (local, abundant)	Diurnal	Medium
Starred agama (*Stellagama stellio*)	Fast	Small (up to 335mm, 1-116g)	Low (non-venomous, harmless)	High (local, abundant)	Diurnal	Low
Common viper (*Daboia palaestinae*)	Fast	Medium-large (160-1360mm,2.3-1800g)	High (highly venomous, potentially fatal)	High (local, abundant)	Mostly nocturnal	Low
Coin-marked snake (*Hemorrhois nummifer*)	Fast	Medium (290-1275mm, 4-560g)	Medium (non-venomous, highly aggressive)	High (local, abundant)	Nocturnal	Low

SI References

1. P. M. Larken, An account of the Zande. *Sudan Notes and Records* 1–55, 85–135 (1926–1927). Available at: https://ehrafworldcultures-yale-edu.ezproxy.haifa.ac.il/cultures/fo07/documents/008 (Accessed: 4 April 2025).
2. J. Beierle, ”Culture summary: Mbuti”. New Haven, Conn.: HRAF. Available at: https://ehrafworldcultures-yale-edu.ezproxy.haifa.ac.il/document?id=fo04-000 (Accessed: 4 April 2025).
3. C. Lévi-Strauss, The Nambicuara. *Bulletin*. *Washington: Smithsonian Institution*, 361–369 (1948). Available at: https://ehrafworldcultures-yale-edu.ezproxy.haifa.ac.il/document?id=sp17-002 (Accessed: 4 April 2025).
4. C. Lévi-Strauss, E. Sittler, *Family and Social Life of the Nambikwara Indians* (Société des Américanistes, Paris, 1948). Avalable at: https://ehrafworldcultures-yale-edu.ezproxy.haifa.ac.il/document?id=sp17-001 (Accessed: 4 April 2025).
5. R. D. Greaves, “Hunting and multifunctional use of bows and arrows: Ethnoarchaeology of technological organization among Pumé hunters of Venezuela” in *Projectile Technology,* H. Knecht, Ed. (Boston, Springer, 1997), pp. 287–320.
6. D. Martínez, J. R Gómez, The use of green iguanas in Fonseca, Colombia. *TRAFFIC Bull.* **25**(2), 73–78 (2013).
7. S. Cane, Australian Aboriginal subsistence in the western desert. *Hum. Ecol.* **15**, 391–434 (1987).
8. R. B. Bird, N. Tayor, B. F. Codding, D. W. Bird, Niche construction and Dreaming logic: aboriginal patch mosaic burning and varanid lizards (*Varanus gouldii*) in Australia. *Proc. R. Soc. B.* **280**(1772), 20132297 (2013).
9. A.T. Elmahi, The spiny-tailed lizard: a constituent of the occasional diet of traditional inland societies in Oman. *Proc. Sem. Arab. Stud.* 31–46 (2002).
10. K. Eilers, W. Koops, H. Udo, H. Van Keulen, J. Noordhuizen, Analysis of *Iguana iguana* farming systems in Nicaragua, Costa Rica and Panama. *Interciencia* **27**(11), 599–606 (2002).
11. D. Natusch, P. W. Aust, C. Caraguel, P. L. Taggart, V. T. Ngo, G. J. Alexander, R. Shine, T. Coulson, Python farming as a flexible and efficient form of agricultural food security. *Sci. Rep.* **14**, 5419 (2024).
12. H. Monchot, S. Bailon, J. Schiettecatte, Archaeozoological evidence for traditional consumption of spiny-tailed lizard (*Uromastyx aegyptia*) in Saudi Arabia. *J. Archaeol. Sci.* **45**, 96–102 (2014).
13. J. Dunne, S. Salvatori, L. Maritan, K. Manning, V. Linseele, T. Gillard, P. Breeze, N. Drake, R. P. Evershed, D. Usai, Wild food: plants, fish and small animals on the menu for early Holocene populations at al-Khiday, Central Sudan. *Afr. Archaeol. Rev.* **39**(3), 255–281 (2022).
14. J.S. Shi, J. Li, Q.P. Yang, N. Liu, X. Chen, Prehistoric snake bones from Zuojiang River Basin, Guangxi, China. *Hist. Biol.* 1-11 (2023).
15. C. Bochaton, R. Boistel, S. Grouard, I. Ineich, A. Tresset, S. Bailon, S, Fossil dipsadid snakes from the Guadeloupe Islands (French West-Indies) and their interactions with past human populations. *Geodiversitas*, **41**(sp2), 501-523 (2019).
16. C. Bochaton, Archaeological occurrences of terrestrial herpetofauna in the insular Caribbean: cultural and biological significance. *R. Soc. Open Sci.* **9**(7), 220256 (2022).
17. E. Tchernov, “The fauna of Sefunim Cave, Mt. Carmel” in *Sefunim Prehistoric Sites Mount Carmel, Israel,* A. Ronen, Ed. (BAR International Series, Oxford), Volume 2, pp. 401–409

18. M. C. Stiner, *The Faunas of Hayonim Cave (Israel): A 200,000-Year Record of Paleolithic Diet, Demography and Society* (Peabody Museum Press, Cambridge, MA, 2005).
19. M. Lev, M. Weinstein-Evron, R. Yeshurun, Squamate bone taphonomy: A new experimental framework and its application to the Natufian zooarchaeological record. *Sci. Rep.* **10**, 9373 (2020).
20. R. Biton, S. Bailon, M. Birkenfeld, A. Bridault, H. Khalaily, F. R. Valla, R. Rabinovich, The anurans and squamates assemblage from Final Natufian Eynan (Ain Mallaha, Israel) with an emphasis on snake-human interactions. *PloS One* **16**, e0247283 (2021).
21. M. Lev, D. Nadel, M. Weinstein-Evron, R. Yeshurun, Squamates and amphibians from the Natufian cemetery of Raqefet Cave, Israel: taphonomy, paleoenvironments and paleoclimate. *Hist. Biol.* **34**(12), 2395–2414 (2022).
22. M. Lev, R. Shimelmitz, M. Weinstein-Evron, R. Yeshurun, Paleoenvironments and climate at Nahal Me'arot (Mount Carmel, Israel) during the Middle and Late Pleistocene: The herpetofauna of Tabun Cave and el-Wad Terrace. *Quat. Sci. Rev.* **307**, 108060 (2023).
23. Y. Katzenelson, Regional climates of Israel. *Meteorological Notes," A/23. (*Beit Dagan, in Hebrew*,* 1967).
24. M. Zohary, *Plant Life of Palestine* (The Roland Press Company, New York, 1962).
25. M. Zohary, *Vegetation of Israel and Adjacent Areas* (Reichert, 1982).
26. Z. Naveh, "Vegetation of the Carmel and Nahal Sefunim and the evolution of the cultural landscape" in *Sefunim Prehistoric Sites, Mount Carmel, Israel*, A. Ronen, Ed. (BAR Publishing, Oxford, 1984), pp. 23–63.
27. M. Weinstein-Evron, *Early Natufian el-Wad Revisited* (Études et Recherches Archéologiques de l'Université de Liège, Liège, 1998).
28. V. Caracuta, M. Weinstein-Evron, R. Yeshurun, D. Kaufman, A. Tsatskin, E. Boaretto, Charred wood remains in the Natufian sequence of el-Wad terrace (Israel): New insights into the climatic, environmental and cultural changes at the end of the Pleistocene. *Quat. Sci. Rev.* **131**, 20–32 (2016).
29. C. Belli, V. Caracuta, D. Nadel, E. Boaretto, R. Yeshurun, M. Weinstein-Evron, Archaeobotany of el-Wad Terrace, Mount Carmel (Israel): Insights into plant exploitation along the Natufian sequence. *Vegetat. Hist. Archaeobot.* **32**(5), 453–474 (2023).
30. M. Weinstein-Evron, R. Yeshurun, H. Ashkenazy, R. Chasan, D. Rosenberg, N. Bachrach, E. Boaretto, V. Caracuta, D. Kaufman, After 80 years–deeper in the Natufian layers of el-Wad Terrace, Mount Carmel, Israel. *Mitekufat Haeven: J. Isr. Prehist. Soc.* **48**, 5–61 (2018).
31. R. Yeshurun, E. Boaretto, M. Lev, M. Orbach, C. Ujma, S. Lavy-Elbaz, L. Amos, M. Weinstein-Evron, A Natufian demographic cycle at el-Wad Terrace, Israel: The rise and fall of the architectural compound. *Archaeol. Res. Asia* **41**, 100599 (2025).
32. R. Yeshurun, G. Bar-Oz, M. Weinstein-Evron, Intensification and sedentism in the terminal Pleistocene Natufian sequence of el-Wad Terrace (Israel). *J. Hum. Evol.* **70**, 16–35 (2014b).
33. D.M.A. Bate, "Paleontology: the fossil fauna of the Wadi el-Mughara caves" In *The Stone Age of Mount Carmel. Vol. I. Excavations at the Wadi Mughar*a, D.A.E. Garrod, D.M.A. Bate, Eds. (Clarendon Press, Oxford, 1937) pp. 137-240.
34. R. Yeshurun, "Paleolithic animal remains in the Mount Carmel caves: A review of the historical and modern research" in *Bones and Identity: Zooarchaeological Approaches to Reconstructing Social and Cultural Landscapes in Southwest Asia*, N. Marom, R. Yeshurun, L. Weissbrod, G. Bar-Oz, Eds. (Oxbow, Oxford, 2016), pp.1–24.

35. G. Bar-Oz, *Epipaleolithic Subsistence Strategies in the Levant: A Zooarchaeological Perspective* (Brill, Boston, 2004).
36. R. Yeshurun, G. Bar-Oz, M. Weinstein-Evron, The role of foxes in the Natufian economy: a view from Mount Carmel, Israel. *Before Farming* **2009**, 1 (2009).
37. L. Weissbrod, T. Dayan, D. Kaufman, M. Weinstein-Evron, Micromammal taphonomy of el-Wad Terrace, Mount Carmel, Israel: distinguishing cultural from natural depositional agents in the Late Natufian. *J. Archaeol. Sci.* **32**(1), 1-17 (2005).
38. L. Weissbrod, G. Bar-Oz, R. Yeshurun, M. Weinstein-Evron, Beyond fast and slow: The mole rat *Spalax ehrenbergi* (order Rodentia) as a test case for subsistence intensification of complex Natufian foragers in southwest Asia. *Quat. Int.* **264**, 4–16 (2012).
39. L. Amos, M. Weinstein-Evron, R. Yeshurun, Seasonal exploitation of migratory waterfowl at Natufian el-Wad Terrace, Mount Carmel, Israel. *Int. J. Osteoarchaeol.* **35**, 555–570 (2025).
40. N. D Munro, Zooarchaeological measures of hunting pressure and occupation intensity in the Natufian: Implications for agricultural origins. *Curr. Anthropol.* 45(4), 5–34 (2004).
41. Y. Fernandez-Jalvo, P. Andrews, *Atlas of Taphonomic Identifications: 1001+ Images of Fossil and Recent Mammal Bone Modification* (Springer, 2016).
42. A. Bar, G. Haimovitch, S. Meiri, *Field guide to the amphibians and reptiles of Israel*. (Frankfurt am Main, 2021).
43. GBIF.org (22 November 2023) GBIF Occurrence Download https://doi.org/10.15468/dl.7u4m3g
44. GBIF.org (22 November 2023) GBIF Occurrence Download https://doi.org/10.15468/dl.jsrvbw
45. GBIF.org (22 November 2023) GBIF Occurrence Download https://doi.org/10.15468/dl.muey34
46. GBIF.org (22 November 2023) GBIF Occurrence Download https://doi.org/10.15468/dl.pn9jj8
47. GBIF.org (22 November 2023) GBIF Occurrence Download https://doi.org/10.15468/dl.xdp4q2
48. GBIF.org (22 November 2023) GBIF Occurrence Download https://doi.org/10.15468/dl.pbpdzg
49. GBIF.org (22 November 2023) GBIF Occurrence Download https://doi.org/10.15468/dl.6qy95n
50. GBIF.org (22 November 2023) GBIF Occurrence Download https://doi.org/10.15468/dl.gfcpvk
51. GBIF.org (22 November 2023) GBIF Occurrence Download https://doi.org/10.15468/dl.3ztuhx
52. GBIF.org (22 November 2023) GBIF Occurrence Download https://doi.org/10.15468/dl.emrssf
53. GBIF.org (22 November 2023) GBIF Occurrence Download https://doi.org/10.15468/dl.bkevsy
54. GBIF.org (22 November 2023) GBIF Occurrence Download https://doi.org/10.15468/dl.kexnsn
55. GBIF.org (22 November 2023) GBIF Occurrence Download https://doi.org/10.15468/dl.r542kv
56. GBIF.org (22 November 2023) GBIF Occurrence Download https://doi.org/10.15468/dl.dwq4w9
57. GBIF.org (22 November 2023) GBIF Occurrence Download https://doi.org/10.15468/dl.f8txj4
58. GBIF.org (22 November 2023) GBIF Occurrence Download https://doi.org/10.15468/dl.m67v9g
59. GBIF.org (5 December 2023) GBIF Occurrence Download https://doi.org/10.15468/dl.fxnnze
60. GBIF.org (5 December 2023) GBIF Occurrence Download https://doi.org/10.15468/dl.sd739x

61. GBIF.org (6 December 2023) GBIF Occurrence Download https://doi.org/10.15468/dl.2au42u
62. GBIF.org (6 December 2023) GBIF Occurrence Download https://doi.org/10.15468/dl.uawbcn
63. GBIF.org (12 December 2023) GBIF Occurrence Download https://doi.org/10.15468/dl.n4zsgk
